# Pregnancy in women with autoimmune diseases and cardiovascular risk in the offspring: a Swedish population-based cohort study

**DOI:** 10.1093/eurheartj/ehag316

**Published:** 2026-04-22

**Authors:** Xingbo Mo, Imre Janszky, Elizabeth V Arkema, Hui Wang, Jette Möller, Yajun Liang, Krisztina D László

**Affiliations:** Department of Global Public Health, Karolinska Institutet, Solnavägen 1E, Stockholm 113 65, Sweden; Department of Epidemiology, School of Public Health, Jiangsu Key Laboratory of Preventive and Translational Medicine for Geriatric Diseases, MOE Key Laboratory of Geriatric Diseases and Immunology, Suzhou Medical College of Soochow University, Renai Road 199, Suzhou 215123, China; Department of Global Public Health, Karolinska Institutet, Solnavägen 1E, Stockholm 113 65, Sweden; Department of Public Health and Nursing, Norwegian University of Science and Technology, Trondheim, Norway; Department of Medicine Solna, Division of Clinical Epidemiology, Karolinska Institutet, Stockholm, Sweden; Department of Global Public Health, Karolinska Institutet, Solnavägen 1E, Stockholm 113 65, Sweden; MOE-Shanghai Key Laboratory of Children’s Environmental Health, Xin Hua Hospital Affiliated to Shanghai Jiao Tong University School of Medicine, Shanghai, China; Department of Global Public Health, Karolinska Institutet, Solnavägen 1E, Stockholm 113 65, Sweden; Department of Global Public Health, Karolinska Institutet, Solnavägen 1E, Stockholm 113 65, Sweden; Department of Global Public Health, Karolinska Institutet, Solnavägen 1E, Stockholm 113 65, Sweden; Department of Public Health and Caring Sciences, Uppsala University, Uppsala, Sweden

**Keywords:** Maternal autoimmune disease, Offspring cardiovascular disease, Pregnancy, Intrauterine exposure, Cohort study, Negative control

## Abstract

**Background and Aims:**

Maternal autoimmune diseases (AIDs) have been linked to adverse birth outcomes that may potentially increase cardiovascular disease (CVD) risk in offspring. However, whether maternal AIDs is associated with offspring CVD remains largely unexplored. This study aimed to examine whether intrauterine exposure to maternal AIDs is associated with offspring CVD up to early adulthood.

**Methods:**

This nationwide population-based cohort study included 1 455 645 live singleton births during 2001–2014 in Sweden and followed them to 31 December 2023. Associations between maternal AIDs and offspring CVD were evaluated using Cox proportional hazard models. Cousin analyses and analyses with paternal AID were conducted to assess shared genetic and familial environmental confounding.

**Results:**

During a median follow-up of 15.2 years (range, 0–23.7 years), 90 046 (6.2%) participants were exposed to maternal AIDs and 40 260 (2.8%) were diagnosed with CVD. Intrauterine exposure to maternal AIDs was associated with an increased risk of any CVD in offspring (hazard ratio 1.19, 95% confidence interval 1.14–1.24). Elevated CVD risks in offspring were observed across maternal AID categories. Paternal AIDs had a weaker association with offspring CVD than maternal AIDs; the associations in the cousin analyses persisted, though they were somewhat weaker than those in the main analyses.

**Conclusions:**

Maternal AIDs is associated with a moderately increased risk of CVD in childhood and early adulthood in offspring. These findings need to be interpreted with caution, as shared familial factors may partly contribute to the observed associations, and further studies are needed to clarify the mechanisms underlying the observed associations.

## Structured Graphical Abstract

**Structured Graphical Abstract:**
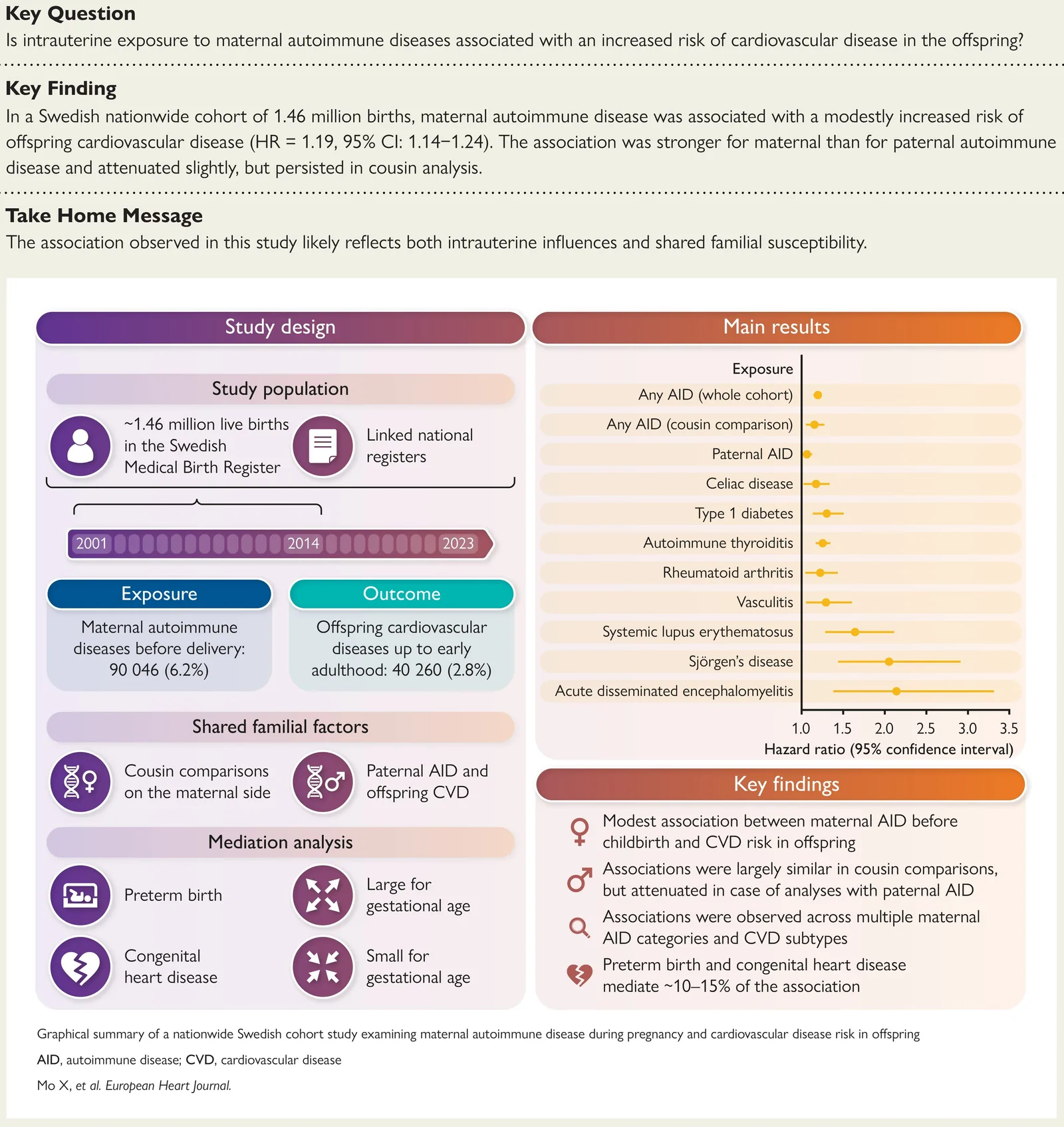

**See the editorial comment for this article ‘Maternal autoimmune diseases and offspring cardiovascular disease: work in progress', by E. Aiton and L. Ghiradi, https://doi.org/10.1093/eurheartj/ehag558.**

## Introduction

Cardiovascular disease (CVD) remain the leading cause of morbidity and mortality worldwide and substantial contributors to the global disease burden. While traditional risk factors such as smoking, obesity, hypertension, and dyslipidaemia are well-established risk factors in adulthood, increasing evidence suggests that the origins of CVD may be traced back to early life, including the intrauterine period.^1^ It is increasingly recognized that *in utero* exposures, including those involving inflammation and immune dysregulation, may predispose the offspring to chronic conditions such as CVD later in life.^2,3^ Epidemiological studies have linked various adverse maternal conditions during pregnancy, such as maternal CVD,^4^ obesity,^5,6^ hypertension,^7^ preeclampsia,^8^ diabetes,^9^ polycystic ovary syndrome,^10^ and stress,^11,12^ to increased risks of CVD in offspring. These associations are thought to be mediated through epigenetic modifications, immune activation, impaired placental function, preterm birth, and alterations in foetal growth and cardiac development.^1^

Autoimmune diseases (AIDs), marked by chronic systemic inflammation and immune dysregulation, affect up to 10% of women of reproductive age.^13,14^ Maternal AIDs during pregnancy have been associated with adverse birth outcomes such as placental dysfunction, preterm birth, and foetal growth restriction.^15–22^ These adverse birth outcomes have, in turn, been linked to an increased cardiovascular risk in offspring.^23–26^ Furthermore, population-based studies have reported increased risks of congenital heart disease in children born to mothers with several AIDs, including systemic lupus erythematosus (SLE),^27–29^ type 1 diabetes (T1D),^30–32^ inflammatory bowel disease,^33^ and celiac disease.^34^ Congenital heart disease is a major contributor to CVD in childhood and early adulthood.^35^ In addition to intrauterine mechanisms, common genetic susceptibility, i.e. that certain AIDs and CVD share overlapping genetic risk loci and immune-related pathways, may also explain an association.^36,37^ However, evidence on the association between maternal AID and long-term cardiovascular outcomes in offspring remains very limited. Direct associations with offspring CVD have been reported only for a limited number of maternal autoimmune conditions, e.g. T1D^9^ and SLE.^27^ Whether other maternal AIDs are associated with a broader spectrum of early onset cardiovascular outcomes, such as vascular diseases, arrhythmias, and structural heart diseases, in offspring remains largely unknown.

In this Swedish nationwide population-based cohort study, we analysed whether intrauterine exposure to maternal AIDs is associated with increased risks of (i) overall CVD, (ii) vascular diseases, (iii) arrhythmias, and (iv) structural heart diseases in offspring.

## Methods

### Study population

For this population-based cohort study, we linked data from multiple Swedish national registers. Our study population included all live singletons born between 1 January 2001 and 31 December 2014 included in the Swedish Medical Birth Register (MBR) (*n* = 1 455 645). These children were followed from birth until the first diagnosis of any CVD, emigration (with migration data available through 2014), death, or the end of follow-up on 31 December 2023, whichever occurred first. The unique personal identity number assigned to all Swedish residents allowed the individual-level linkage across national registers. The registers linked for the present study included the MBR (including >98% of all births in Sweden, 1973–2014), the National Patient Register (NPR, established in 1964, with nationwide inpatient coverage since 1987 and nationwide coverage of specialized non-private outpatient care since 2001), the Cause of Death Register (virtually complete since 1952), the Total Population Register (1968–2014), the Multigeneration Register (1961–2014), and the Education Register (1990–2014). These registers have been shown to have high coverage and validity in epidemiological research.^38–41^

### Variables

#### Exposure assessment

Data on maternal AIDs were retrieved from the NPR (inpatient and outpatient records) and from the MBR. Maternal intrauterine AIDs exposure was defined as a diagnosis of any autoimmune condition prior to or during the index pregnancy. From the NPR, we retrieved information on primary and secondary AIDs diagnoses of the mother recorded before delivery. In the MBR, we searched for up to 12 maternal diagnoses that are registered at discharge from delivery based on information recorded during pregnancy and at delivery. The International Classification of Diseases (ICD) codes (ICD-8, ICD-9, and ICD-10 revisions) used to define AIDs are presented in the Supplementary data online, *Table S1*. We defined exposure both as a composite variable (any AIDs) and by specific disease groups and types, the latter depending on statistical power. Nine groups of AIDs were defined, i.e. endocrine AID,s inflammatory arthritis, vasculitis, connective tissue AIDs, skin AID, blood AIDs, nervous system AIDs, gastrointestinal AIDs, and other AIDs, using the classification of Song *et al*.^42^ Previous validation studies of the register indicated high accuracy for diagnoses of several AIDs in the NPR, with positive predictive values around 90% for rheumatoid arthritis and multiple sclerosis, and approximately 70%–90% for inflammatory bowel disease subtypes.^38,43–45^

#### Outcome definitions

The study outcome was diagnosis of CVD in offspring, identified using ICD-10 codes from the NPR (both primary and secondary diagnoses from inpatient and outpatient settings, see Supplementary data online, *Table S1*) and the Cause of Death Register (main cause of death). We evaluated the following outcomes: (i) overall CVD; (ii) vascular diseases, including ischaemic heart disease, cerebrovascular disease, hypertension, and venous thromboembolism; (iii) arrhythmias; and (iv) structural heart diseases, including cardiomyopathy, heart failure, and valvular heart disease. Validation studies of cardiovascular diagnoses in Sweden reported positive predictive values around 90% for major CVD categories such as ischaemic heart disease, stroke, heart failure, and atrial fibrillation.^38,46–49^

#### Covariates

Covariates were selected based on prior literature, biological plausibility, and a directed acyclic graph (see Supplementary data online, *Figure S1*); we distinguished confounders assessed at baseline from potential mediators.^4,42,50,51^ Our main covariates were calendar year of birth and maternal sociodemographic characteristics, age at delivery, country of birth, parity, marital status, educational level and household income in the year of childbirth. Information on maternal country of birth and marital status was obtained from the Total Population Register. Information on the level of education was obtained from the Swedish Register of Education, whereas data on household income were extracted from the Register of Incomes and Taxes. From the MBR, we also retrieved information on age at delivery, parity, body mass index (BMI), and smoking in early pregnancy. Additional maternal health-related factors, i.e. CVD and psychiatric disorders before childbirth, were retrieved from the NPR. Pre-gestational diabetes and pre-gestational hypertension were identified using data from both the MBR and the NPR. Mothers’ first-degree relatives (i.e. parents and siblings) were identified through the Swedish Multigeneration Register. Family history of CVD for the mothers was defined based on information from the NPR.

Preterm birth was defined as gestational age <37 weeks at birth. Small for gestational age (SGA) and large for gestational age (LGA) were defined as birth weight below the 10th percentile and above the 90th percentile according to the Swedish reference curve, respectively.^52^ Information on these variables was obtained from the MBR. Placental abruption and preeclampsia were also identified using diagnosis records in the MBR. Diagnoses of congenital heart disease in offspring were also obtained from the NPR. The corresponding ICD codes are shown in Supplementary data online, *Table S1*.

All variables were categorized as shown in *Table 1*.

**Table 1:** Characteristics of the exposed and unexposed groups at birth

Study variables	Total population	Exposed to maternal autoimmune diseases	
	(*n* = 1 455 645)	No (*n* = 1 365 599)	Yes (*n* = 90 046)
Offspring sex			
Male	748 328 (51.41)	702 190 (51.42)	46 138 (51.24)
Female	707 317 (48.59)	663 409 (48.58)	43 908 (48.76)
Calendar year of birth			
2001–2005	477 105 (32.78)	459 201 (33.63)	17 904 (19.88)
2006–2010	534 778 (36.74)	502 194 (36.77)	32 584 (36.19)
2011–2014	443 762 (30.49)	404 204 (29.60)	39 558 (43.93)
Preterm birth			
No	1 368 986 (94.05)	1 287 200 (94.26)	81 786 (90.83)
Yes	86 022 (5.91)	77 801 (5.70)	8221 (9.13)
Missing	637 (0.04)	598 (0.04)	39 (0.04)
Birth weight for gestational age			
Appropriate	1 146 017 (78.73)	1 079 419 (79.04)	66 598 (73.96)
Small	148 424 (10.20)	138 930 (10.17)	9494 (10.54)
Large	157 816 (10.84)	144 073 (10.55)	13 743 (15.26)
Missing	3388 (0.23)	3177 (0.23)	211 (0.23)
Congenital heart disease			
No	1 422 834 (97.75)	1 335 388 (97.79)	87 446 (97.11)
Yes	32 811 (2.25)	30 211 (2.21)	2600 (2.89)
Major malformation			
No	1 358 706 (93.34)	1 275 426 (93.40)	83 280 (92.49)
Yes	96 939 (6.66)	90 173 (6.60)	6766 (7.51)
Offspring autoimmune diseases			
No	1 392 795 (95.68)	1 308 512 (95.82)	84 283 (93.60)
Yes	62 850 (4.32)	57 087 (4.18)	5763 (6.40)
Mother’s country of birth			
Sweden	1 139 475 (78.28)	1 064 279 (77.93)	75 196 (83.51)
Other country	314 488 (21.60)	299 757 (21.95)	14 731 (16.36)
Missing	1682 (0.12)	1563 (0.11)	119 (0.13)
Maternal age at the time of the index birth (years)			
≤19	22 476 (1.54)	21 669 (1.59)	807 (0.90)
20–24	184 523 (12.68)	176 241 (12.91)	8282 (9.20)
25–29	432 067 (29.68)	408 766 (29.93)	23 301 (25.88)
30–34	763 469 (52.45)	710 920 (52.06)	52 549 (58.36)
≥35	53 110 (3.65)	48 003 (3.52)	5107 (5.67)
Maternal education in the year of the index birth			
Primary and lower secondary	157 405 (10.81)	148 814 (10.90)	8591 (9.54)
Upper secondary	580 432 (39.87)	545 094 (39.92)	35 338 (39.24)
Bachelor or higher	686 150 (47.14)	640 881 (46.93)	45 269 (50.27)
Missing	31 658 (2.17)	30 810 (2.26)	848 (0.94)
Maternal income in the year of the index birth			
Low	464 155 (31.89)	440 632 (32.27)	23 523 (26.12)
Middle	473 433 (32.52)	444 481 (32.55)	28 952 (32.15)
Higher	517 762 (35.57)	480 194 (35.16)	37 568 (41.72)
Missing	295 (0.02)	292 (0.02)	3 (0)
Maternal marital status in the year of the index birth			
Yes	516 893 (35.51)	486 694 (35.64)	30 199 (33.54)
No	844 259 (58.0)	791 678 (57.97)	52 581 (58.39)
Missing	94 493 (6.49)	87 227 (6.39)	7266 (8.07)
Maternal body mass index in early pregnancy (kg/m^2^)			
Underweight (<18.5)	31 991 (2.20)	30 160 (2.21)	1831 (2.03)
Normal weight (18.5–24.9)	803 603 (55.21)	756 825 (55.42)	46 778 (51.95)
Overweight (25.0–29.9)	328 414 (22.56)	307 397 (22.51)	21 017 (23.34)
Obesity (≥30.0)	157 178 (10.80)	144 257 (10.56)	12 921 (14.35)
Missing	134 459 (9.24)	126 960 (9.30)	7499 (8.33)
Parity of the index birth			
1	638 334 (43.85)	600 997 (44.01)	37 337 (41.46)
2	533 354 (36.64)	499 364 (36.57)	33 990 (37.75)
≥3	283 957 (19.51)	265 238 (19.42)	18 719 (20.79)
Maternal smoking in early pregnancy			
No	1 288 495 (88.52)	1 208 352 (88.49)	80 143 (89.00)
Yes	103 355 (7.10)	97 308 (7.13)	6047 (6.72)
Missing	63 795 (4.38)	59 939 (4.39)	3856 (4.28)
Maternal pre-gestational hypertension			
No	1 390 273 (95.51)	1 307 006 (95.71)	83 267 (92.47)
Yes	65 372 (4.49)	58 593 (4.29)	6779 (7.53)
Maternal pre-gestational diabetes			
No	1 431 935 (98.37)	1 352 130 (99.01)	79 805 (88.63)
Yes	23 710 (1.63)	13 469 (0.99)	10 241 (11.37)
Preeclampsia			
No	1 410 670 (96.91)	1 325 163 (97.04)	85 507 (94.96)
Yes	44 975 (3.09)	40 436 (2.96)	4539 (5.04)
Placental abruption			
No	1 450 232 (99.63)	1 360 599 (99.63)	89 633 (99.54)
Yes	5413 (0.37)	5000 (0.37)	413 (0.46)
Gestational age at birth (weeks), mean (standard deviation)	39.7 (1.94)	39.7 (1.03)	39.3 (2.18)
Maternal psychiatric disorders before childbirth			
No	1 312 447 (90.16)	1 236 682 (90.56)	75 765 (84.14)
Yes	143 198 (9.84)	128 917 (9.44)	14 281 (15.86)
Maternal cardiovascular diseases before childbirth			
No	1 332 849 (91.56)	1 255 338 (91.93)	77 511 (86.08)
Yes	122 796 (8.44)	110 261 (8.07)	12 535 (13.92)
Mother’s family history of cardiovascular disease			
No	753 309 (51.75)	708 385 (51.87)	44 924 (49.89)
Yes	456 191 (31.34)	421 936 (30.90)	34 255 (38.04)
Missing	246 145 (16.91)	235 278 (17.23)	10 867 (12.07)

Data are presented as *n* (%), unless otherwise indicated.

### Statistical analysis

We analysed the association between maternal AIDs during pregnancy and offspring CVD using Cox proportional hazard models. We ran three models, Model 1 was a crude model with offspring age as the underlying time scale; Model 2 was adjusted for age (as the time scale), offspring birth year, parity, maternal country of birth, education, and marital status at delivery; Model 3 was additionally adjusted for maternal CVD and psychiatric disorders before childbirth, pre-gestational diabetes, pre-gestational hypertension. As the additional covariates included in Model 3 may lie on the causal pathway or represent early manifestations of shared cardiometabolic vulnerability, and their role as confounders or mediators is uncertain, to consider concerns about potential over-adjustment, Model 3 was specified as a separate, exploratory model rather than as an estimate of the total causal effect. We conducted stratified analyses by offspring sex, offspring age, household income, maternal CVD, diabetes, and hypertension before delivery. The proportional hazards assumption was assessed for the Cox model by visual inspection of log-minus-log survival curve, which suggested approximately parallel trend (see Supplementary data online, *Figure S2*); however, as Schoenfeld residuals suggested a violation of the proportional hazard assumption, we utilized flexible parametric survival models to estimate the time-varying effects of maternal any AID before birth on risk of overall CVD in offspring.

#### Mediation analysis

Furthermore, we performed mediation analyses to assess whether preterm birth, SGA, LGA, or congenital heart disease—all three known to be associated both with maternal AIDs during pregnancy and CVD in offspring^15–25,27,28,35^—mediated the observed associations. The mediation analyses were conducted within the counterfactual framework introduced by Valeri and VanderWeele^53^ and by using the CMAverse package in R.^54^ The mediation models adjusted for offspring’s calendar year of birth, parity, maternal country of birth, age, education, income, and marital status in the year of childbirth, and maternal psychiatric diseases before childbirth, based on a prespecified causal diagram (see Supplementary data online, *Figure S3*). When there was evidence of exposure-mediator interaction, we additionally applied the four-way decomposition method described by VanderWeele to disentangle the total effect into the controlled direct effect, pure indirect effect, reference interaction, and mediated interaction, thereby allowing preterm birth to act simultaneously as a mediator and an effect modifier.^55^

#### Family-based comparisons

We performed analyses with several family-based designs. First, we examined the association between paternal AIDs (defined using both primary and secondary diagnoses from the NPR) diagnosed before the index child’s birth and offspring CVD, as a negative control analysis, to assess the potential role of the shared genetic and familial factors and to help distinguish intrauterine effects from residual confounding.^56,57^ To account for assortative mating and to ensure appropriate interpretation of the negative-control design, in the paternal models, we adjusted for maternal AIDs in addition to variables in Models 1–3.^58^ The difference between the maternal-offspring and paternal-offspring CVD associations was quantified and statistically tested using the Wald test. Second, to account for shared familial confounding, we also performed cousin-comparison analyses among offspring of maternal siblings. We fitted stratified Cox proportional hazards models with cousin sets as strata, thereby comparing exposed and unexposed cousins within the same extended family while controlling for measured covariates.

#### Sensitivity analysis

We conducted several sensitivity analyses to assess the robustness of our findings. First, we adjusted for additional variables available only in a subset of the cohort, including maternal smoking in early pregnancy, maternal BMI in early pregnancy, and family history of CVD. Second, we adjusted for preeclampsia and placental abruption, two placentation disorders that could be affected by maternal AIDs, but in the case of which evidence regarding a link with offspring CVD is currently limited to justify a formal mediation analysis. Third, we adjusted for length of gestation, a variable that may affect both the probability of being diagnosed with AIDs and the offspring risk of CVD, but which could also be influenced by AIDs. Fourth, we repeated all main analyses after excluding children with congenital heart disease, major malformations, and any AIDs. Fifth, to explore the potential importance of the timing of exposure, we categorized maternal AIDs according to the interval between diagnosis (of the first AIDs in the NPR) and childbirth into three mutually exclusive groups: no maternal AIDs (reference group), maternal AIDs diagnosis ≤2, and maternal AIDs diagnosis >2 years before delivery. Sixth, to explore the importance of undiagnosed or subclinical AIDs in the studied association, we performed analyses with maternal AIDs diagnosed within three years postpartum, as these may reflect conditions already present but undetected during pregnancy. Seventh, to increase exposure specificity, we conducted sub-analyses by (i) restricting exposure definition to mothers with at least two recorded diagnoses for a specific AID in national registers, and (ii) restricting to mothers diagnosed with only one type of AID, to evaluate the effect of isolated disease versus multi-system involvement. Eighth, we repeated our main analyses using alternative definitions of the outcomes. Specifically, we first required at least two recorded ICD-10 diagnoses for the same cardiovascular condition to increase diagnostic specificity. In addition, we applied outcome-specific definitions, i.e. vascular diseases were identified using primary inpatient diagnoses and the underlying cause of death; arrhythmias were defined using both primary and secondary diagnoses from inpatient and outpatient records; and structural heart diseases were identified using primary diagnoses from both inpatient and outpatient settings. Finally, to assess the robustness of the observed associations to potential unmeasured confounding, we also calculated *E*-values following the approach described by VanderWeele and Ding.^59^

All analyses were conducted using R version 4.3.1.

### Patient and public involvement

Patients or members of the public were not involved in the design, conduct, reporting, or dissemination plans of this study. The study was based entirely on anonymized population-based registry data.

## Results

A total of 1 455 645 participants were included in the final analyses, of whom 90 046 (6.2%) were exposed to maternal AIDs (*Table 1*). Compared with unexposed children, those exposed to maternal AIDs were born in later parts of the study period, were more frequently preterm (9.1% vs 5.7%), had higher rates of congenital heart disease (2.9% vs. 2.2%), and were more likely to be LGA at birth (15.3% vs. 10.6%). Children of mothers with AIDs were more likely to be exposed to maternal obesity (14.4% vs. 10.6%), hypertension (7.5% vs. 4.3%), pre-gestational diabetes (11.4% vs. 1.0%), preexisting CVD (13.9% vs. 8.1%), and a family history of CVD (38.0% vs. 30.9%).

### Maternal autoimmune disease and offspring cardiovascular risk

Offspring were followed from birth up to a maximum age of 23.7 years, with a median follow-up age of 15.2 years (interquartile range, 11.8–18.9 years). During the follow-up, 6007 (0.4%) offspring were censored due to non-CVD death, and 30 531 (2.1%) due to emigration. The cumulative incidence of CVD was higher in offspring exposed to maternal AID than in the unexposed group (*Figure 1*). In age-specific absolute risk analyses, offspring exposed to maternal AID had consistently higher cumulative incidences of overall and subtype-specific CVD across childhood, adolescence, and young adulthood; however, the absolute risk differences compared with unexposed offspring were small (see Supplementary data online, *Table S2*). Over the 22 114 121 person-years of follow-up, we observed 40 260 incident cases of CVD in the offspring. Maternal AIDs diagnosed before or during pregnancy was associated with an increased risk of overall CVD in offspring. The incidence rate of overall CVD was 21.3 per 10 000 person-years among offspring exposed to maternal AID before birth and 18.0 per 10 000 person-years among unexposed offspring, corresponding to an absolute difference of approximately three additional cases per 10 000 person-years (*Figure 2*). The hazard ratio (HR) for overall CVD was 1.19 [95% confidence interval (CI) 1.14–1.24] in the adjusted model (Model 3). The flexible parametric model showed that the HR for offspring CVD associated with maternal AIDs was slightly higher during early follow-up, decreased over the first years, and remained relatively stable thereafter (see Supplementary data online, *Figure S4*).

**Figure 1:**
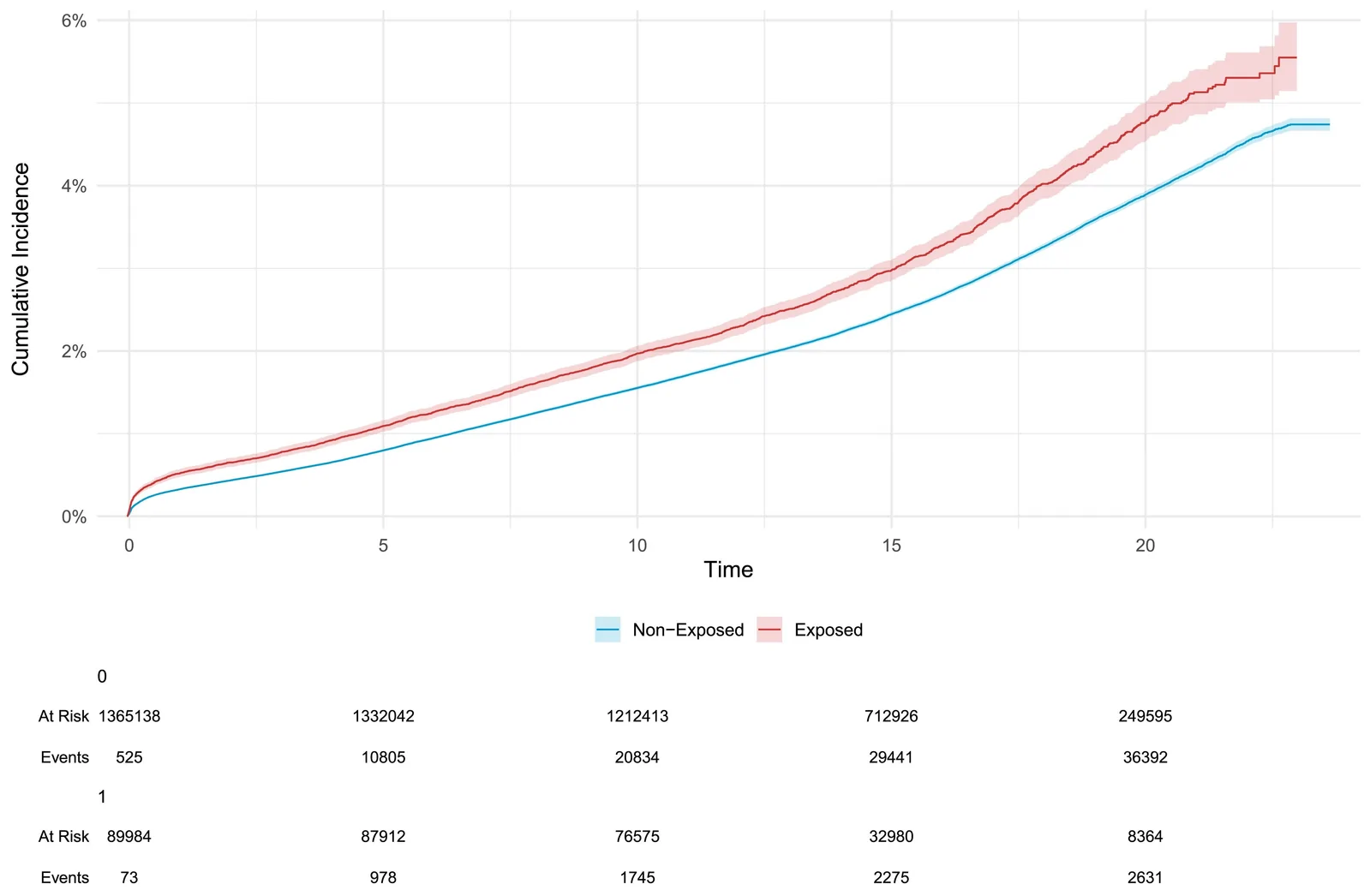
Unadjusted cumulative incidence of any cardiovascular disease in offspring by exposure to any maternal autoimmune diseases

**Figure 2:**
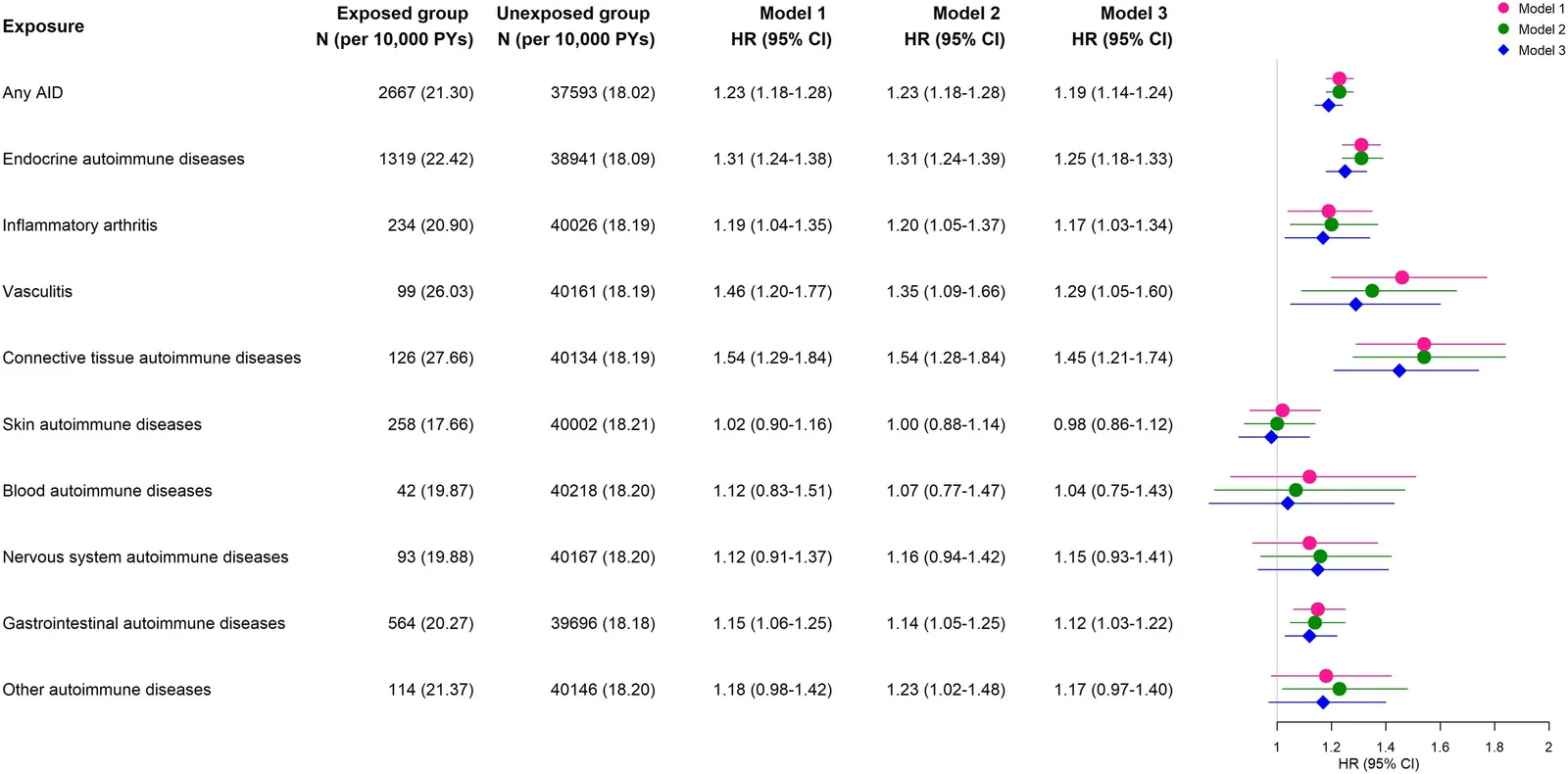
Hazard ratios and 95% confidence intervals for any cardiovascular diseases in offspring according to maternal autoimmune diseases during pregnancy. AID, autoimmune disease; CI, confidence interval; HR, hazard ratio; PY, person-year. Models 1 were adjusted for age at follow-up; Models 2 were additionally adjusted for offspring’s calendar year of birth, maternal country of birth, parity, education, income, and marital status in the year of birth; Models 3 were adjusted for factors in Model 2 and for maternal cardiovascular and psychiatric diseases before childbirth, maternal pre-gestational hypertension, and pre-gestational diabetes

When exploring associations by categories of maternal AIDs, we observed an increased risk of CVD in offspring across several major AID groups, including, endocrine AID (HR 1.25, 95% CI 1.18–1.33), inflammatory AID (HR 1.17, 95% CI 1.03–1.34), vasculitis (HR 1.29, 95% CI 1.05–1.60), connective tissue AID (HR 1.45, 95% CI 1.21–1.74), and gastrointestinal AID (HR 1.12, 95% CI 1.03–1.22) (*Figure 2*). We also found associations between several specific AIDs and offspring CVD, with Sjögren’s disease, SLE, and acute disseminated encephalomyelitis having the highest effect sizes (*Figure 3*). Analyses by specific categories of CVD in offspring showed that maternal AIDs during pregnancy was associated with increased risks of vascular diseases (HR 1.18, 95% CI 1.05–1.33), including ischaemic heart disease (HR 1.70, 95% CI 1.03–2.79) and hypertension (HR 1.23, 95% CI 1.04–1.45), arrhythmias (HR 1.19, 95% CI 1.08–1.30), and structural heart diseases (HR 1.23, 95% CI 1.08–1.40) (*Figure 4*). Some specific AIDs were also associated with the three major analysed CVD categories, i.e. vascular diseases, arrhythmias, and structural heart diseases; however, estimates were often imprecise due to lower statistical power (see Supplementary data online, *Table S3*).

**Figure 3:**
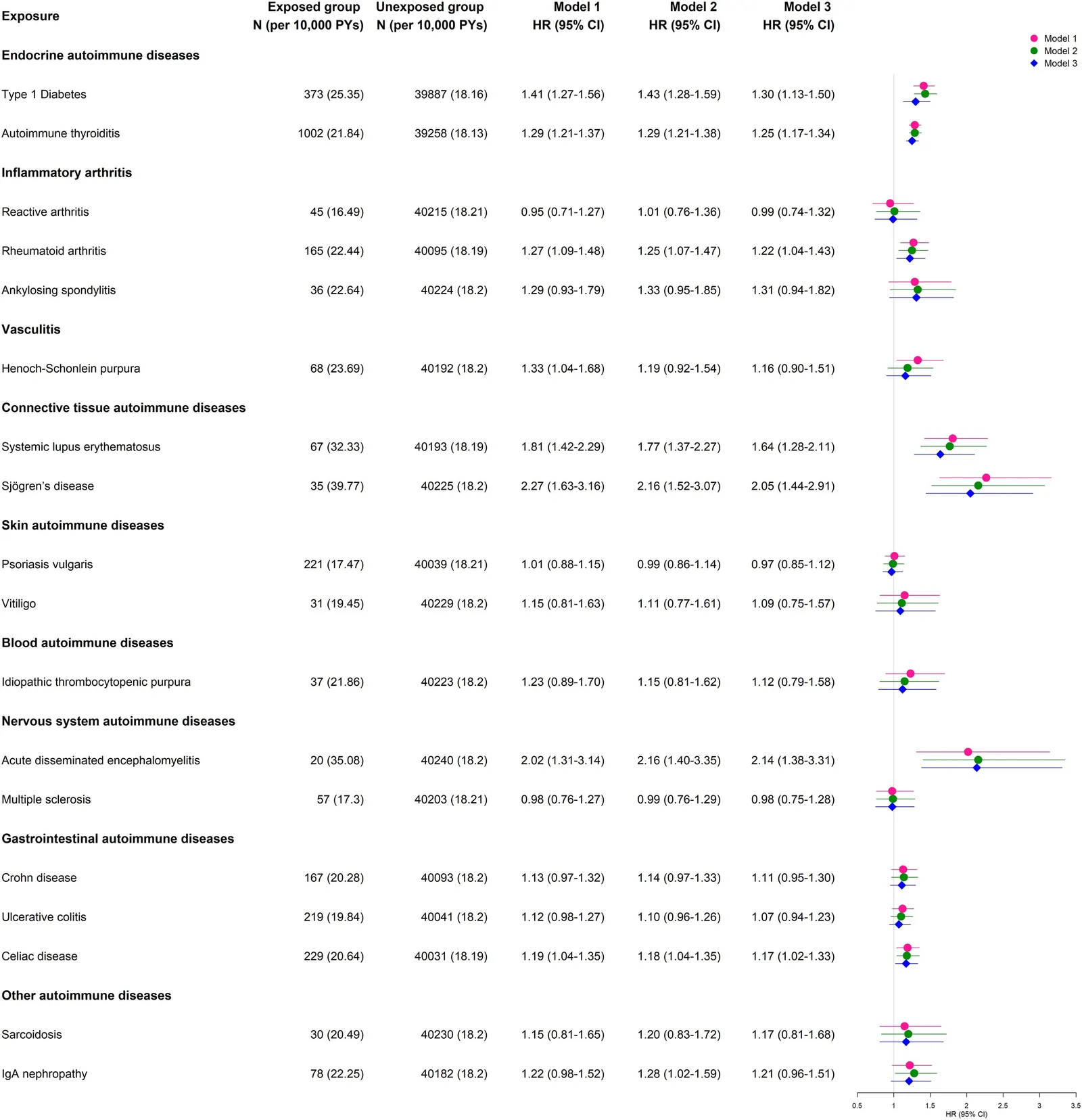
Hazard ratios and 95% confidence intervals for any cardiovascular diseases in offspring according to maternal-specific autoimmune diseases during pregnancy. CI, confidence interval; HR, hazard ratio; PY, person-year. Only autoimmune diseases with ≥20 cardiovascular disease events occurring in the exposed group were included in our analyses. Models 1 were adjusted for age at follow-up; Models 2 were additionally adjusted for offspring’s calendar year of birth, maternal country of birth, parity, education, income, and marital status in the year of birth; Models 3 were adjusted for factors in Model 2 and for maternal cardiovascular and psychiatric diseases before childbirth, maternal pre-gestational hypertension, and pre-gestational diabetes

**Figure 4:**
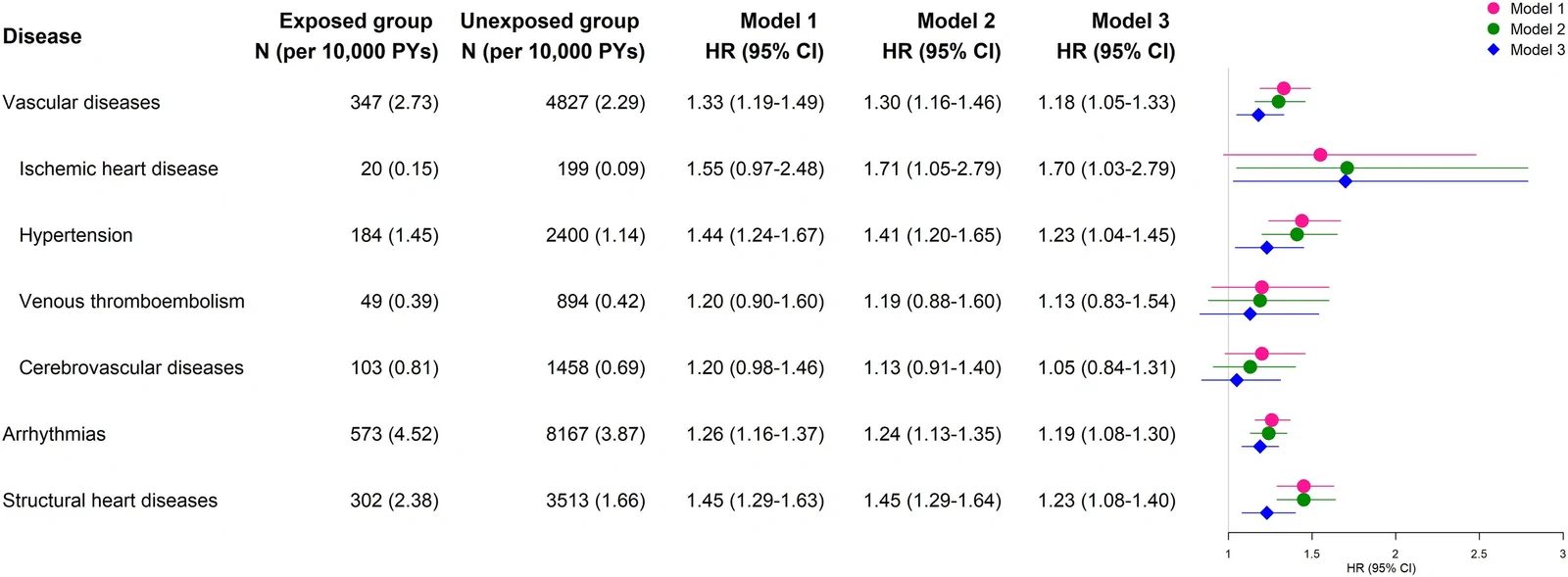
Hazard ratios and 95% confidence intervals for cardiovascular disease categories in offspring according to any maternal autoimmune disease during pregnancy. CI, confidence interval; HR, hazard ratio; PY, person-year. Models 1 were adjusted for age at follow-up; Models 2 were additionally adjusted for offspring’s calendar year of birth, maternal country of birth, parity, education, income, and marital status in the year of birth; Models 3 were adjusted for factors in Model 2 and for maternal cardiovascular and psychiatric diseases before childbirth, maternal pre-gestational hypertension, and pre-gestational diabetes

There was no support in the data for a strong association between paternal AIDs during the pregnancy and offspring CVD; point estimates were small and more modest than those corresponding to maternal exposure (*Table 2*). The formal statistical test suggested that the association corresponding to maternal AIDs was stronger than that corresponding to paternal AIDs, with a ratio of HR of 1.13 (95% CI 1.06–1.22), as assessed using a Wald test (*P* = 0.0006). In cousin-comparison analyses, the association between maternal AIDs and offspring CVD was somewhat attenuated compared with the main cohort analysis, although point estimates remained above unity (*Table 3*).

**Table 2:** Hazard ratios and 95% confidence intervals for cardiovascular diseases in offspring according to paternal autoimmune diseases during the partner’s pregnancy

Disease	Events (per 10 000 PYs)		HR (95% CI)			Ratio^a^
	Exposed	Unexposed	Model 1	Model 2	Model 3	
Any cardiovascular diseases	1328 (18.90)	38,932 (18.18)	0.98 (0.93–1.04)	1.07 (1.01–1.13)	1.05 (0.99–1.11)	1.13 (1.06–1.22)
Vascular diseases	177 (2.49)	4997 (2.31)	1.02 (0.88–1.19)	1.14 (0.98–1.32)	1.08 (0.92–1.28)	1.09 (0.89–1.34)
Hypertension	96 (1.35)	2488 (1.15)	1.11 (0.91–1.37)	1.27 (1.03–1.56)	1.20 (0.96–1.50)	1.03 (0.78–1.35)
Venous thromboembolism	26 (0.37)	917 (0.42)	0.82 (0.55–1.21)	1.01 (0.68–1.50)	0.96 (0.63–1.48)	1.18 (0.69–1.99)
Cerebrovascular diseases	51 (0.72)	1510 (0.70)	0.98 (0.74–1.29)	1.00 (0.75–1.33)	0.98 (0.72–1.33)	1.07 (0.73–1.56)
Stroke	38 (0.53)	1157 (0.53)	0.95 (0.69–1.31)	0.98 (0.71–1.35)	0.93 (0.65–1.33)	1.20 (0.78–1.86)
Ischaemic stroke	25 (0.35)	664 (0.31)	1.09 (0.73–1.63)	1.11 (0.74–1.66)	1.08 (0.70–1.67)	1.21 (0.71–2.06)
Arrhythmias	287 (4.04)	8453 (3.90)	0.98 (0.87–1.10)	1.06 (0.94–1.20)	1.07 (0.94–1.21)	1.11 (0.95–1.30)
Structural heart diseases	137 (1.93)	3678 (1.70)	1.08 (0.91–1.28)	1.16 (0.98–1.38)	1.07 (0.89–1.29)	1.15 (0.92–1.44)
Heart failure	25 (0.35)	797 (0.37)	0.91 (0.61–1.35)	0.99 (0.66–1.48)	0.90 (0.58–1.40)	1.11 (0.65–1.90)

CI, confidence interval; HR, hazard ratio; PY, person-year.

Only autoimmune diseases with ≥20 cardiovascular disease events occurring in the exposed group were included in our analyses.

Models 1 were adjusted for age at follow-up; Models 2 were additionally adjusted for maternal country of birth, education, income, and marital status in the year of birth; Models 3 were adjusted for factors in Model 2 and for maternal cardiovascular and psychiatric diseases before childbirth, maternal pre-gestational hypertension, and pre-gestational diabetes.

^a^The ratio of maternal-to-paternal associations was calculated as the ratio of the adjusted HR for maternal autoimmune disease to the corresponding adjusted HR for paternal autoimmune disease; CIs were derived using a Wald test.

**Table 3:** Hazard ratios and 95% confidence intervals for any cardiovascular disease in offspring according to maternal autoimmune diseases during pregnancy in cousin analyses

Exposure	Events (per 10 000 PYs)		HR (95% CI)		
	Exposed	Unexposed	Model 1	Model 2	Model 3
Any autoimmune diseases	1521 (21.18)	21 222 (18.01)	1.21 (1.11–1.32)	1.19 (1.09–1.31)	1.15 (1.05–1.27)
Endocrine autoimmune diseases	707 (22.83)	22 036 (18.07)	1.30 (1.14–1.48)	1.29 (1.12–1.48)	1.22 (1.06–1.41)
Inflammatory arthritis	131 (19.37)	22 612 (18.18)	1.03 (0.77–1.37)	1.01 (0.75–1.37)	1.00 (0.74–1.36)
Vasculitis	59 (25.31)	22 684 (18.17)	1.34 (0.83–2.16)	1.17 (0.70–1.97)	1.14 (0.68–1.93)
Connective tissue autoimmune diseases	76 (27.43)	22 667 (18.17)	1.40 (0.95–2.08)	1.46 (0.97–2.20)	1.41 (0.93–2.13)
Skin autoimmune diseases	152 (17.46)	22 591 (18.19)	0.99 (0.77–1.28)	0.94 (0.72–1.24)	0.94 (0.72–1.23)
Blood autoimmune diseases	27 (22.24)	22 716 (18.18)	0.91 (0.48–1.72)	0.83 (0.43–1.62)	0.80 (0.41–1.55)
Nervous system autoimmune diseases	62 (22.19)	22 681 (18.18)	1.49 (0.97–2.29)	1.49 (0.95–2.33)	1.49 (0.95–2.34)
Gastrointestinal autoimmune diseases	341 (19.32)	22 402 (18.17)	1.14 (0.95–1.37)	1.11 (0.91–1.35)	1.08 (0.89–1.31)
Other autoimmune diseases	72 (22.43)	22 671 (18.18)	1.34 (0.86–2.07)	1.32 (0.83–2.09)	1.27 (0.80–2.01)

CI, confidence interval; HR, hazard ratio; PY, person-year.

Only autoimmune diseases with ≥20 cardiovascular disease events occurring in the exposed group were included in our analyses.

Models 1 were adjusted for age at follow-up; Models 2 were additionally adjusted for maternal country of birth, education, income, and marital status in the year of birth; Models 3 were adjusted for factors in Model 2 and for maternal cardiovascular and psychiatric diseases before childbirth, maternal pre-gestational hypertension, and pre-gestational diabetes.

Subgroup analyses revealed no clear evidence for interactions between maternal AIDs before childbirth and offspring sex, offspring age, household income, maternal CVD, or maternal hypertension on overall CVD risk in offspring (see Supplementary data online, *Figure S5*–*S9*). However, the association between maternal AIDs and structural heart diseases in offspring was stronger among offspring of mothers with diabetes compared with those of mothers without diabetes (see Supplementary data online, *Figure S10*).

When exploring the mediators of the studied association, maternal AIDs were associated with increased risks of preterm birth, SGA, and LGA, and congenital heart disease in offspring, while preterm birth, SGA, and congenital heart disease were in turn associated with higher risks of offspring CVD (see Supplementary data online, *Table S4*). Accordingly, preterm birth and congenital heart disease in offspring partially mediated the association between maternal AIDs and offspring CVD, accounting for an estimated 9.05% and 14.55% of the effect, respectively (see Supplementary data online, *Table S5*). In additional four-way decomposition analyses accounting for exposure-mediator interaction, preterm birth contributed to the association through both mediation and interaction (*P* = 0.03), with the pure indirect effect, reference interaction, and mediated interaction accounting for 5.8% (95% CI 4.30–8.10), 7.8% (95% CI 1.0–15.5), and 3.3% (95% CI 0.4–6.6) of the excess relative risk, respectively.

### Sensitivity analyses

Adjusting for maternal smoking (see Supplementary data online, *Table S6*), BMI (see Supplementary data online, *Table S7*), family history of CVD in the subset of study participants with available data on these measures (see Supplementary data online, *Table S8*), and for preeclampsia, placental abruption, and gestational age at delivery (see Supplementary data online, *Table S9*) yielded similar estimates to those from the main models. Exclusion of offspring with congenital heart disease, major malformations, or any AIDs did not substantially change the associations (see Supplementary data online, *Table S10*–12). When categorizing maternal AIDs by timing of diagnosis relative to childbirth, HRs for offspring cardiovascular outcomes were generally higher for maternal diagnoses recorded within 2 years before delivery compared with earlier diagnoses, although the magnitude of associations varied across specific disease types and outcomes (see Supplementary data online, *Table S13*). The associations remained consistent with those in the main analyses when, to capture preclinical disease, in the definition of exposure, we included only maternal AIDs diagnosed within three years postpartum (see Supplementary data online, *Table S14*). Analyses defining exposure based on at least two recorded maternal diagnoses for the same AID resulted in similar results to those in the main models (see Supplementary data online, *Table S15*). We observed similar results to those observed in the main analyses when exposure was defined based on only one type of maternal AID (see Supplementary data online, *Table S16*). Finally, results remained consistent when using alternative outcome definitions (see Supplementary data online, *Tables S17* and *S18*). The *E*-values ranged from 1.24 to 2.26 across AID categories (see Supplementary data online, *Table S19*).

## Discussion

In this large population-based cohort study of 1 455 645 children born in Sweden between 2001 and 2014, we found that maternal AIDs diagnosed prior to delivery was associated with an increased risk of early onset CVD in offspring. The associations were evident for several specific AIDs and across specific subtypes of CVD, including vascular diseases, arrhythmias, and structural heart diseases. These associations remained robust across multiple sensitivity analyses and were partly mediated by preterm birth and congenital heart disease (*Structured Graphical Abstract*).

### Comparison with previous studies

Our findings build on and extend the limited literature linking a few specific maternal AID during pregnancy to long-term cardiovascular outcomes in offspring. A large Danish cohort study reported that maternal T1D was associated with increased CVD risk in offspring.^9^ Another study found that children born to mothers with SLE had an elevated risk of arrhythmic disorders and cardiomyopathy during the follow-up until the age of 13 years.^27^ To the best of our knowledge, no prior large-scale, comprehensive study has examined long-term cardiovascular outcomes in children exposed *in utero* to a wide range of maternal AIDs. In line with the two previously mentioned studies,^9,27^ we found that maternal T1D and SLE were associated with increased risks of overall CVD and several CVD subtypes in offspring. Beyond these two well-established autoimmune conditions, we identified novel associations involving a broader spectrum of maternal AIDs affecting multiple organ systems—including endocrine, inflammatory arthritis, vasculitis, connective tissue, and gastrointestinal—and elevated risks of CVD in offspring. Specifically, maternal diagnoses of autoimmune thyroiditis, rheumatoid arthritis, Sjögren’s disease, acute disseminated encephalomyelitis, coeliac disease, and IgA nephropathy were all linked to increased offspring CVD risk. Notably, our findings suggested that maternal diabetes may modify the association between maternal AIDs and offspring CVD risk, especially structural CVD. Previous studies have consistently reported that maternal diabetes is associated with an increased risk of cardiovascular outcomes in offspring.^9^ This observation may reflect additional effects beyond the simple combination of maternal inflammation and metabolic disturbances, although the interpretation is limited by the heterogeneous definition of maternal diabetes, which included T1D; further studies are needed to disentangle diabetes subtypes and confirm these interactions. Our study thus significantly extends the current understanding of the long-term cardiovascular consequences of *in utero* exposure to maternal autoimmunity.

### Potential explanations for the findings

Several mechanisms may underlie the observed associations between maternal AIDs and the increased cardiovascular risk in offspring. First, many AIDs identified in our study to be associated with CVD, such as T1D, SLE, rheumatoid arthritis, and Sjögren’s disease, are characterized by chronic systemic inflammation and immune dysregulation. These maternal conditions may impair placental development, disrupt oxygen and nutrient transfer, and induce epigenetic alterations in the fetus.^60–63^ Such intrauterine insults align with the ‘foetal programming’ hypothesis and may predispose offspring to structural heart diseases and vascular dysfunction.^64^ In line with this, our mediation analysis suggested that preterm birth and congenital heart disease partly mediated the association between maternal AIDs and offspring CVD, supporting the role of placental dysfunction, impaired cardiac morphogenesis, and shortened gestation in this pathway. However, the overall proportion mediated was relatively small, suggesting that additional pathways beyond these commonly considered birth outcomes may contribute and that these mediation estimates should be interpreted with caution. Second, pharmacologic treatments commonly used to manage AIDs—such as corticosteroids, non-steroidal anti-inflammatory drugs, and immunosuppressants—may interfere with foetal cardiovascular development as these medications can impact foetal haemodynamic, myocardial cell proliferation, and maternal-foetal immune tolerance.^65–68^ Third, some AIDs may exert specific maternal physiologic alterations that affect foetal cardiovascular development. For example, T1D and IgA nephropathy can alter maternal metabolic and renal homeostasis, leading to abnormal placental perfusion or foetal exposure to metabolic by-products, thereby impairing vascular development.^69,70^ Similarly, coeliac disease may contribute to maternal micronutrient deficiencies and systemic inflammation, both of which are known to adversely affect foetal cardiovascular development.^71^ Finally, certain AIDs may directly impact foetal cardiac conduction.^72^ In our study, SLE, T1D, and autoimmune thyroiditis were associated with arrhythmia risk in offspring. These conditions may affect foetal electrophysiology through mechanisms such as systemic inflammation, transplacental transfer of maternal autoantibodies (e.g. anti-Ro/SSA in SLE), metabolic disturbances (e.g. maternal hyperglycaemia in T1D), or thyroid hormone imbalances.^73–75^ These disruptions may impair maturation of the foetal cardiac conduction system, increasing susceptibility to arrhythmic outcomes.

Our findings for some more common AIDs suggest that not all maternal AIDs may confer an increased CVD risk to offspring. For instance, in our study, psoriasis vulgaris and multiple sclerosis—both relatively prevalent conditions—were apparently not associated with offspring CVD. One possible explanation is that these conditions may involve more localized or organ-specific inflammation, with limited systemic immune activation or placental involvement during pregnancy.^76,77^ This could result in relatively lower foetal exposure to inflammatory cytokines or metabolic disturbances, thereby reducing the likelihood of long-term cardiovascular effects. However, as this is the first study to investigate the association between maternal AIDs during pregnancy and the offspring's CVD risk, our findings require replication in other large cohorts.

Beyond intrauterine mechanisms, several pre- and post-natal familial and environmental factors, including shared genetic susceptibility and family lifestyle, may also contribute to offspring cardiovascular risk. These pathways could not be fully evaluated in our study and as in any observational study, residual confounding cannot be ruled out. However, to be able to fully explain our results, any potential remaining confounder would need to be relatively strongly associated with both maternal AIDs and CVD risk in the offspring (see the *E*-values for the minimal associations) and be largely independent of the covariates included in our models. Family-based comparisons can help distinguish intrauterine effects from shared familial influences. In our study, the data were not compatible with a strong association between paternal AIDs during pregnancy and offspring CVD, whereas maternal AIDs was consistently associated with increased CVD risk in offspring. Because paternal AIDs are not expected to affect the intrauterine environment, the near absence of a paternal association argues against shared genetic or familial environmental factors as the primary explanation, and instead supports a role for pregnancy-specific or intrauterine mechanisms. In the cousin comparison analyses, effect estimates were attenuated compared with the main analyses. This pattern is consistent with a partial contribution of shared familial factors, including genetic susceptibility and early-life environmental influences to the association between maternal AIDs and offspring CVD. It should be noted that cousins share only a proportion of genetic (approximately 25%) and familial environmental factors, thus, our analyses could only partially control for these factors. Taken together, these complementary analyses are consistent with a role of both the shared environmental and genetic factors and pregnancy-specific or intrauterine mechanisms in the observed associations. This interpretation underscores the importance of cautious causal inference and highlights the complexity of disentangling biological and familial mechanisms in intergenerational studies.

### Strengths and limitations

This study has several strengths. It leverages a large, nationwide birth cohort with virtually complete long-term follow-up enabled by high-quality Swedish health registers. The use of both population-based and family-based designs enhances the robustness of the findings and helps account for shared familial factors. Furthermore, the inclusion of a wide range of AIDs and cardiovascular outcomes allowed for a comprehensive assessment of associations across various disease categories.

Nevertheless, several limitations should be acknowledged. First, although we adjusted for a wide range of maternal and familial characteristics in multivariable models and by using cousin analyses and analyses with AIDs in partners as partner negative controls, residual confounding—particularly by unmeasured genetic, environmental, or behavioural factors—cannot be entirely ruled out. Second, misclassification of exposure and outcome is possible, especially since subclinical or mild AIDs might not have been captured by the NPR. This misclassification is likely to be non-differential and to result in a downwardly biased estimate. A differential detection of offspring CVD may be possible in case of CVDs that may be asymptomatic, if mothers with AIDs seek medical care for their children more frequently than their healthy counterparts, leading to increased surveillance of their children. Third, data on maternal disease activity, subtypes (e.g. SSA/SSB + Sjögren’s disease, seropositive/seronegative rheumatoid arthritis) and specific medication use during pregnancy were not comprehensively available in the registers, limiting our ability to explore dose–response relationships or to disentangle the effects of underlying autoimmune condition from its treatment. Fourth, we had wide CIs in the case of some less common AIDs. Consequently, we cannot have a firm conclusion regarding those conditions. Also, subgroup analyses for rare AIDs or specific cardiovascular subtypes may have been underpowered, and those results should be interpreted with caution. Further studies with larger samples and longer follow-up are needed to validate these findings and refine risk estimates. Fifth, information on maternal family history of CVD was incomplete. In addition, emigration data were available only through 2014. Based on the emigration data available through 2014, the annual emigration rate among offspring was low and declined steadily over time, from approximately 0.21% in 2001% to 0.01% in 2014. Therefore, we do not expect this to substantially affect the results. Sixth, a potential live birth bias may exist, as foetuses exposed to maternal AIDs who did not survive to birth could not be included, potentially leading to a biased estimation of the true association. Finally, while our study benefits from the comprehensive and high-quality nature of Swedish national data, the generalizability of our findings to other populations with different genetic backgrounds, healthcare systems, or AIDs prevalence may be limited.

## Conclusions

Maternal AIDs diagnosed before childbirth were associated with a modestly increased risk of CVD in offspring up to young adulthood. These findings may highlight the potential long-term cardiovascular implications of maternal immune health during pregnancy and may underscore the importance of monitoring and support for children born to mothers with autoimmune conditions. However, the attenuation of associations in family-based analyses suggests that shared familial factors may at least partly explain these findings. Therefore, the results should be interpreted cautiously. Further studies are needed to disentangle intrauterine effects from familial confounding and to clarify the biological pathways underlying these associations.

## Supplementary Material

ehag316_Supplementary_Data

## Declarations

### Data Availability

In accordance with Swedish data protection legislation and General Data Protection Regulation, individual-level register data are not publicly accessible. Researchers based in Sweden may request access to comparable datasets following approval from a Swedish regional ethics review authority and subsequent authorization from the data owners. Applications can be submitted to the Swedish National Board of Health and Welfare and to Statistics Sweden through their respective data access services.
