# Supplementary material for: Pregnancy in women with autoimmune diseases and cardiovascular risk in the offspring: a Swedish population-based cohort study
Source: Eur Heart J. 2026 Apr 22;47(39):5822–36. doi: 10.1093/eurheartj/ehag316 (PMC13690822; doi:10.1093/eurheartj/ehag316)
Supplement: Supplementary file 1 [file ehag316_supplementary_data.docx]

**Supplementary Online Content**

Table S1. The International Classification of Diseases codes used to identify the diagnoses from the Patient Register, Cause of Death Register or Medical Birth Register

| Disease | | ICD version | | | | | |  |
| --- | --- | --- | --- | --- | --- | --- | --- | --- |
|  |  | ICD-8 | | ICD-9 | | ICD-10 | |  |
| **Any cardiovascular diseases** | | 390-458 | | 390-549 | | I00-I99 | |  |
| Valvular heart disease | | 394-396 | | 394-396 | | I06, I34-I35 | |  |
| Hypertension | | 400-404 | | 401-405, 642A, 642B, 642C 642H | | I10-I15, O10, O11 | |  |
| Maternal pre-gestational and gestational hypertensive disorder | | 400-404, 637.01, 637.03, 637.04, 637.09, 637.99, 637.10 | | 401-405, 642A, 642B, 642C, 642D, 642E, 642F, 642G, 642H, 642X | | I10-I15, O10, O11, O13, O14, O15, O16 | |  |
| Ischemic heart disease | | 410-414 | | 410-414 | | I20-I23 | |  |
| Arrhythmia | | 427 | | 426, 427 | | I44-I49 | |  |
| Atrial fibrillation | | 427.92 | | 427D | | I48 | |  |
| Heart failure | | 427.00, 427.10 | | 428 | | I11.0, I13.0, I13.2, I50 | |  |
| Cerebrovascular diseases | | 430-438 | | 430-438 | | I61-I69, G45 | |  |
| Stroke | | 430, 431, 433, 434, 436 | | 430, 431, 434, 436 | | I60, I61, I63, I64 | |  |
| Ischemic stroke | | 433, 434 | | 434 | | I63 | |  |
| Hemorrhagic stroke | | 430, 431 | | 430, 431 | | I60, I61 | |  |
| Venous thromboembolism | | 450, 451 | | 415B, 451 | | I26, I80.1, I80.2, I80.3 | |  |
| Congenital heart disease | | 746, 747 | | 745, 746, 747 | | Q20-Q28 | |  |
| Major malformations* | | - | | - | | Q175, Q180, Q181, Q249, Q250, Q270, Q289, Q314, Q315, Q320, Q381, Q523, Q53, Q65, Q665, Q666, Q667, Q668, Q669, Q690, Q699, Q703, Q7550, Q760, Q799, Q825, Q829 | |  |
| Diabetes mellitus | | 250 | | 250, 648A, 648W | | E10-E14, O24 | |  |
| Preeclampsia | | 637.03, 637.04, 637.09, 637.99, 637.10 | | 642E, 642F, 642G | | O14, O15 | |  |
| Placental abruption | | 632.10, 651.4 | | 641C | | O45 | |  |
| Psychiatric disorders | | 290-315 | | 290-319 | | F00-F99 | |  |
| **Autoimmune diseases**† | |  | |  | |  | |  |
|  | **Endocrine autoimmune diseases** | |  | |  | |  | |
|  | Type 1 diabetes | | - | | - | | E10 | |
|  | Autoimmune thyroiditis | | 242.00, 242.09, 244, 245.03 | | 242A, 242X, 244W, 244X, 245C, 245W | | E03.5, E03.9, E05.0, E05.5, E05.9, E06.3, E06.5 | |
|  | Primary adrenocortical insufficiency | | 255.1 | | 255E | | E27.1, E27.2 | |
|  | Autoimmune polyglandular syndrome | | 258.1 | | 258B | | E31.0 | |
|  | **Inflammatory arthritis** | |  | |  | |  | |
|  | Reactive arthritis (Reiter’s syndrome) | | 099.3, 711.1 | | 099D, 711A | | M02.3, M02.8, M02.9 | |
|  | Rheumatoid arthritis | | 712.00, 712.10, 712.20, 712.39, 712.38 | | 714A, 714B, 714C, 714D, 719D | | M05, M06, M08.0, M08.1, M08.2, M08.3, M08.4 | |
|  | Ankylosing spondylitis | | 712.4 | | 720A | | M45 | |
|  | **Vasculitis** | |  | |  | |  | |
|  | Polyarteritis nodosa and related condition | | 446 | | 446A, 446B | | M30 | |
|  | Thrombotic microangiopathy | | - | | 446G | | M31.1 | |
|  | Granulomatosis with polyangiitis/Wegener granulomatosis | | 446.2 | | 446E | | M31.3 | |
|  | Microscopic polyangiitis | | - | | - | | M31.7 | |
|  | Henoch-Schonlein purpura | | 287 | | 287A | | D69.0 | |
|  | Polymyalgia rheumatica | | 446.30, 446.31, 446.39,717.9 | | 446F, 725 | | M31.5, M31.6, M35.3 | |
|  | **Connective tissue autoimmune diseases** | |  | |  | |  | |
|  | Systemic lupus erythematosus | | 734.1 | | 710A | | M32 | |
|  | Polymyositis/Dermatopolymyositis | | 716.00, 716.10 | | 710D, 710E | | M33.0, M33.1, M33.2, M33.9 | |
|  | Systematic sclerosis/Scleroderma | | 710.0, 734.0 | | 710B | | M34 | |
|  | Sjögren’s disease | | 734.9 | | 710C | | M35.0 | |
|  | Mixed connective tissue disease | | 734.98, 734.91, 734.99 | | 710X, 710W | | M35.1 | |
|  | Behcet′s syndrome | | 136 | | 136B | | M35.2 | |
|  | **Skin autoimmune diseases** | |  | |  | |  | |
|  | Pemphigus vulgaris | | 694 | | 694E | | L10.0 | |
|  | Bullous pemphigoid | | 694.05 | | 694F | | L12 | |
|  | Dermatitis herpetiformis | | 693.99 | | 694A | | L13.0 | |
|  | Psoriasis vulgaris | | 696 | | 696A, 696B | | L40 | |
|  | Alopecia areata | | 704 | | 704A | | L63 | |
|  | Vitiligo | | 709.05 | | 709A | | L80 | |
|  | **Blood autoimmune diseases** | |  | |  | |  | |
|  | Pernicious anemia | | 281 | | 281A | | D51.0 | |
|  | Autoimmune hemolytic anemia | | 283.90, 283.92 | | 283A | | D59.0, D59.1 | |
|  | Idiopathic thrombocytopenic purpura | | 287.3 | | 287D | | D69.3 | |
|  | **Nervous system autoimmune diseases** | |  | |  | |  | |
|  | Acute disseminated encephalomyelitis | | - | | - | | G04 | |
|  | Anti-NMDA receptor encephalitis | | - | | - | | G13.1 | |
|  | Multiple sclerosis | | 340 | | 340 | | G35 | |
|  | Neuromyelitis optica | | 341 | | 341A | | G36 | |
|  | Guillain-Barré syndrome | | 354.01 | | 357A | | G61.0, G61.1, G61.8, G61.9 | |
|  | Myasthenia gravis | | 733 | | 358A | | G70.0 | |
|  | **Gastrointestinal autoimmune diseases** | |  | |  | |  | |
|  | Primary biliary cirrhosis | | - | | 571G | | K74.3 | |
|  | Crohn disease | | 563 | | 555 | | K50 | |
|  | Ulcerative colitis | | 563.10, 569.02 | | 556 | | K51 | |
|  | Celiac disease | | 269.00, 269.98 | | 579A | | K90.0 | |
|  | **Other autoimmune diseases** | |  | |  | |  | |
|  | Acute rheumatic fever and chorea | | 390, 391, 392 | | 390, 391, 392 | | I00, I01.0, I01.1, I01.2, I01.8, I01.9, I02.0, I02.9 | |
|  | Sarcoidosis | | 135 | | 135 | | D86 | |
|  | Autoimmune hepatitis | | 571.93 | | 571.9 | | K75.4 | |
|  | IgA nephropathy | | 580, 582 | | 580, 582 | | N00, N01, N03, N05 | |

ICD: International Classification of Diseases. The International Classification of Diseases’ 8^th^ revision was used during 1973-1986, the 9^th^ revision during 1987-1996 and the 10^th^ revision since 1997.

*: The ICD-10 codes for major malformations were defined according to Hossin et al. (Eur Heart J, 2024; 7;45(38):4111-4123).

†: The nine groups of autoimmune diseases were defined following the classification used in the paper of Song et al. (JAMA 2018; 319(23):2388-400).

Table S2. Absolute risks at fixed ages in exposed and unexposed offspring

| Disease | Age group | Exposed | |  | Non-exposed | |
| --- | --- | --- | --- | --- | --- | --- |
|  |  | Events | Incidence† |  | Events | Incidence† |
| Any CVD | ≤10 | 1738 | 1.93 |  | 20775 | 1.52 |
|  | 11-18 | 2535 | 2.82 |  | 34117 | 2.50 |
|  | >18 | 2667 | 2.96 |  | 37593 | 2.75 |
| Vascular diseases | ≤10 | 182 | 0.20 |  | 2090 | 0.15 |
|  | 11-18 | 313 | 0.35 |  | 4123 | 0.30 |
|  | >18 | 347 | 0.39 |  | 4827 | 0.35 |
| Ischemic heart disease | ≤10 | 17 | 0.02 |  | 155 | 0.01 |
|  | 11-18 | 19 | 0.02 |  | 181 | 0.01 |
|  | >18 | 19 | 0.02 |  | 199 | 0.01 |
| Cerebrovascular diseases | ≤10 | 79 | 0.09 |  | 942 | 0.07 |
|  | 11-18 | 96 | 0.11 |  | 1339 | 0.10 |
|  | >18 | 103 | 0.11 |  | 1458 | 0.11 |
| Hypertension | ≤10 | 75 | 0.08 |  | 886 | 0.06 |
|  | 11-18 | 169 | 0.19 |  | 2123 | 0.16 |
|  | >18 | 184 | 0.20 |  | 2400 | 0.18 |
| Venous thromboembolism | ≤10 | 13 | 0.0 |  | 160 | 0.01 |
|  | 11-18 | 36 | 0.04 |  | 582 | 0.04 |
|  | >18 | 49 | 0.05 |  | 894 | 0.07 |
| Arrhythmia | ≤10 | 333 | 0.37 |  | 3868 | 0.28 |
|  | 11-18 | 532 | 0.59 |  | 7127 | 0.52 |
|  | >18 | 573 | 0.64 |  | 8167 | 0.60 |
| Structural heart diseases | ≤10 | 234 | 0.26 |  | 2295 | 0.17 |
|  | 11-18 | 286 | 0.32 |  | 3182 | 0.23 |
|  | >18 | 302 | 0.34 |  | 3513 | 0.26 |
| Heart failure | ≤10 | 45 | 0.05 |  | 567 | 0.04 |
|  | 11-18 | 52 | 0.06 |  | 673 | 0.05 |
|  | >18 | 54 | 0.06 |  | 768 | 0.06 |

CVD: Cardiovascular disease.

†: per 10,000 person-years.

Table S3. Hazard ratios and 95% confidence intervals for cardiovascular disease subgroups in offspring according to maternal autoimmune diseases during pregnancy

| Outcome | Exposure | Events (per 10,000 PYs) | |  | HR (95% CI) | | |
| --- | --- | --- | --- | --- | --- | --- | --- |
|  |  | Exposed | Non-exposed |  | Model 1 | Model 2 | Model 3 |
| **Vascular diseases** | **Endocrine autoimmune diseases** | 182 (3.05) | 4992 (2.29) |  | 1.29 (1.09-1.53) | 1.53 (1.31-1.79) | 1.29 (1.09-1.53) |
|  | Type 1 Diabetes | 77 (5.14) | 5097 (2.29) |  | 1.70 (1.23-2.35) | 2.33 (1.85-2.93) | 1.70 (1.23-2.35) |
|  | Autoimmune thyroiditis | 113 (2.43) | 5061 (2.31) |  | 1.17 (0.96-1.42) | 1.25 (1.02-1.51) | 1.17 (0.96-1.42) |
|  | **Inflammatory arthritis** | 31 (2.73) | 5143 (2.31) |  | 1.23 (0.85-1.77) | 1.27 (0.88-1.83) | 1.23 (0.85-1.77) |
|  | Rheumatoid arthritis | 24 (3.22) | 5150 (2.31) |  | 1.40 (0.92-2.13) | 1.46 (0.96-2.22) | 1.40 (0.92-2.13) |
|  | **Skin autoimmune diseases** | 34 (2.30) | 5140 (2.31) |  | 1.02 (0.71-1.46) | 1.05 (0.73-1.50) | 1.02 (0.71-1.46) |
|  | Psoriasis vulgaris | 28 (2.19) | 5146 (2.31) |  | 0.94 (0.63-1.40) | 0.96 (0.64-1.43) | 0.94 (0.63-1.40) |
|  | **Gastrointestinal autoimmune diseases** | 74 (2.63) | 5100 (2.31) |  | 1.14 (0.90-1.46) | 1.17 (0.92-1.49) | 1.14 (0.90-1.46) |
|  | Crohn disease | 24 (2.88) | 5150 (2.31) |  | 1.29 (0.86-1.95) | 1.32 (0.88-1.99) | 1.29 (0.86-1.95) |
|  | Ulcerative colitis | 33 (2.95) | 5141 (2.31) |  | 1.22 (0.84-1.77) | 1.24 (0.86-1.80) | 1.22 (0.84-1.77) |
|  | Celiac disease | 21 (1.87) | 5153 (2.31) |  | 0.82 (0.52-1.29) | 0.84 (0.54-1.32) | 0.82 (0.52-1.29) |
| **Arrhythmias** | **Endocrine autoimmune diseases** | 282 (4.73) | 8458 (3.89) |  | 1.30 (1.14-1.48) | 1.35 (1.19-1.53) | 1.30 (1.14-1.48) |
|  | Type 1 Diabetes | 82 (5.47) | 8658 (3.90) |  | 1.35 (1.00-1.83) | 1.42 (1.13-1.78) | 1.35 (1.00-1.83) |
|  | Autoimmune thyroiditis | 211 (4.55) | 8529 (3.90) |  | 1.30 (1.12-1.50) | 1.34 (1.16-1.55) | 1.30 (1.12-1.50) |
|  | **Inflammatory arthritis** | 47 (4.15) | 8693 (3.91) |  | 1.08 (0.80-1.45) | 1.11 (0.83-1.50) | 1.08 (0.80-1.45) |
|  | Rheumatoid arthritis | 38 (5.10) | 8702 (3.91) |  | 1.29 (0.93-1.80) | 1.34 (0.96-1.87) | 1.29 (0.93-1.80) |
|  | **Vasculitis** | 24 (6.21) | 8716 (3.91) |  | 1.40 (0.91-2.16) | 1.49 (0.97-2.29) | 1.40 (0.91-2.16) |
|  | **Connective tissue autoimmune diseases** | 39 (8.43) | 8701 (3.90) |  | 1.88 (1.34-2.63) | 2.04 (1.46-2.86) | 1.88 (1.34-2.63) |
|  | Systemic lupus erythematosus | 21 (9.94) | 8719 (3.90) |  | 2.04 (1.27-3.29) | 2.27 (1.41-3.65) | 2.04 (1.27-3.29) |
|  | **Skin autoimmune diseases** | 48 (3.25) | 8692 (3.91) |  | 0.83 (0.62-1.13) | 0.85 (0.63-1.16) | 0.83 (0.62-1.13) |
|  | Psoriasis vulgaris | 42 (3.29) | 8698 (3.91) |  | 0.87 (0.63-1.20) | 0.89 (0.65-1.22) | 0.87 (0.63-1.20) |
|  | **Gastrointestinal autoimmune diseases** | 123 (4.37) | 8617 (3.90) |  | 1.08 (0.89-1.30) | 1.11 (0.92-1.34) | 1.08 (0.89-1.30) |
|  | Crohn disease | 32 (3.84) | 8708 (3.91) |  | 0.87 (0.59-1.26) | 0.90 (0.61-1.31) | 0.87 (0.59-1.26) |
|  | Ulcerative colitis | 46 (4.12) | 8694 (3.91) |  | 0.97 (0.71-1.33) | 1.00 (0.74-1.37) | 0.97 (0.71-1.33) |
|  | Celiac disease | 55 (4.90) | 8685 (3.90) |  | 1.30 (0.99-1.71) | 1.33 (1.01-1.75) | 1.30 (0.99-1.71) |
|  | **Other autoimmune diseases** | 22 (4.07) | 8718 (3.91) |  | 0.99 (0.64-1.51) | 1.06 (0.69-1.63) | 0.99 (0.64-1.51) |
| **Structural heart diseases** | **Endocrine autoimmune diseases** | 184 (3.09) | 3631 (1.67) |  | 1.42 (1.19-1.68) | 1.87 (1.60-2.18) | 1.42 (1.19-1.68) |
|  | Type 1 Diabetes | 96 (6.43) | 3719 (1.67) |  | 2.38 (1.74-3.25) | 3.90 (3.17-4.81) | 2.38 (1.74-3.25) |
|  | Autoimmune thyroiditis | 106 (2.28) | 3709 (1.69) |  | 1.24 (1.01-1.51) | 1.35 (1.11-1.66) | 1.24 (1.01-1.51) |
|  | **Inflammatory arthritis** | 20 (1.76) | 3795 (1.70) |  | 1.03 (0.66-1.62) | 1.08 (0.69-1.69) | 1.03 (0.66-1.62) |
|  | **Skin autoimmune diseases** | 29 (1.96) | 3786 (1.70) |  | 1.12 (0.77-1.64) | 1.16 (0.79-1.69) | 1.12 (0.77-1.64) |
|  | Psoriasis vulgaris | 27 (2.11) | 3788 (1.70) |  | 1.20 (0.81-1.78) | 1.24 (0.84-1.84) | 1.20 (0.81-1.78) |
|  | **Gastrointestinal autoimmune diseases** | 46 (1.63) | 3769 (1.71) |  | 1.01 (0.76-1.36) | 1.05 (0.78-1.40) | 1.01 (0.76-1.36) |
|  | Celiac disease | 25 (2.22) | 3790 (1.70) |  | 1.35 (0.91-2.00) | 1.42 (0.96-2.10) | 1.35 (0.91-2.00) |

CI: Confidence interval; HR: Hazard ratio; PY: Person-year.

Only autoimmune diseases with ≥ 20 cardiovascular disease events occurring in the exposed group were included in our analyses.

Models 1 were adjusted for age at follow-up; Models 2 were adjusted for offspring’s calendar year of birth, maternal country of birth, parity, education, income, and marital status in the year of childbirth; Models 3 were adjusted for factors in model 2 and for maternal cardiovascular and psychiatric diseases before childbirth, maternal pre-gestational hypertension, and pre-gestational diabetes.

Table S4. The exposure-mediator and mediator-outcome associations

| Mediator | Exposure (maternal AID) | Outcome (offspring CVD) |
| --- | --- | --- |
|  | OR (95% CI) | HR (95% CI) |
| Preterm birth | 1.41 (1.38-1.45) | 1.52 (1.47-1.57) |
| Small for gestational age | 1.10 (1.08-1.13) | 1.22 (1.19-1.26) |
| Large for gestational age | 1.18 (1.16-1.21) | 1.02 (0.99-1.06) |
| Congenital heart disease | 1.20 (1.15-1.26) | 7.20 (6.99-7.41) |

AID: Autoimmune disease; CI: Confidence interval; CVD: cardiovascular diseases; HR: Hazard ratio; OR: Odds ratio.

Analyses were adjusted for offspring’s calendar year of birth, maternal country of birth, parity, age, education, income, and marital status in the year of childbirth, maternal cardiovascular and psychiatric diseases before childbirth, maternal pre-gestational hypertension, and pre-gestational diabetes.

Table S5. Hazard ratios and 95% confidence intervals for any cardiovascular disease according to any maternal autoimmune disease during pregnancy mediated by preterm birth, small or large for gestational age, and congenital heart disease

| Mediator | Number of participants | HR (95% CI) | | | Proportion mediated (%) | *P* value for the interaction† |
| --- | --- | --- | --- | --- | --- | --- |
|  |  | Total effect | TNDE | TNIE |  |  |
| Preterm birth | 1,129,756 | 1.18 (1.13-1.23) | 1.17 (1.12-1.23) | 1.01 (1.01-1.02) | 9.05 (5.93-13.54) | 0.03 |
| Small for gestational age | 999,880 | 1.18 (1.12-1.24) | 1.18 (1.12-1.23) | 1.00 (1.00-1.01) | 3.22 (1.54-5.52) | 0.06 |
| Large for gestational age | 1,020,774 | 1.17 (1.12-1.23) | 1.17 (1.12-1.22) | 1.00 (1.00-1.00) | 1.24 (-0.21-3.27) | 0.21 |
| Congenital heart disease | 1,130,256 | 1.18 (1.13-1.24) | 1.16 (1.10-1.21) | 1.02 (1.02-1.03) | 14.55 (10.16-21.07) | 0.18 |

CI: Confidence interval; HR: Hazard ratio; TNDE: Total natural direct effect; TNIE: Total natural indirect effect (Mediated effect).

Analyses were adjusted for offspring’s calendar year of birth, maternal country of birth, parity, age, education, income, and marital status in the year of childbirth, maternal psychiatric diseases before childbirth.

The numbers of participants vary among analyses corresponding to different mediators because of the differences in the number of individual with missing data on each mediator and exposure-mediator interactions.

† *P* value for the exposure-mediator interactions. Wald tests were used to calculate two-sided *P* values.

Table S6. Hazard ratios and 95% confidence intervals for cardiovascular diseases in offspring according to maternal autoimmune diseases during pregnancy in individuals who had data on maternal smoking in early pregnancy (n= 1,391,850)

| Outcome | Exposure | Events (per 10,000 PYs) | |  | HR (95% CI) | |
| --- | --- | --- | --- | --- | --- | --- |
|  |  | Exposed | Non-exposed |  | Model 1 | Model 2 |
| **Any CVD** | **Any AID** | 2498 (20.88) | 35,652 (17.88) |  | 1.18 (1.13-1.23) | 1.18 (1.13-1.23) |
|  | **Endocrine autoimmune diseases** | 1241 (22.09) | 36,909 (17.94) |  | 1.25 (1.17-1.33) | 1.25 (1.17-1.33) |
|  | Type 1 Diabetes | 349 (25.21) | 37,801 (18.01) |  | 1.31 (1.14-1.51) | 1.31 (1.14-1.51) |
|  | Autoimmune thyroiditis | 945 (21.48) | 37,205 (17.98) |  | 1.25 (1.16-1.33) | 1.25 (1.16-1.33) |
|  | **Inflammatory arthritis** | 220 (20.61) | 37,930 (18.04) |  | 1.16 (1.01-1.33) | 1.16 (1.01-1.33) |
|  | Reactive arthritis | 43 (16.54) | 38,107 (18.06) |  | 1.00 (0.74-1.35) | 1.00 (0.74-1.35) |
|  | Rheumatoid arthritis | 154 (22.01) | 37,996 (18.04) |  | 1.20 (1.02-1.42) | 1.20 (1.02-1.42) |
|  | Ankylosing spondylitis | 34 (22.18) | 38,116 (18.05) |  | 1.29 (0.92-1.82) | 1.29 (0.92-1.82) |
|  | **Vasculitis** | 93 (25.59) | 38,057 (18.04) |  | 1.27 (1.02-1.58) | 1.27 (1.02-1.58) |
|  | Henoch-Schonlein purpura | 64 (23.28) | 38,086 (18.05) |  | 1.14 (0.87-1.49) | 1.14 (0.87-1.49) |
|  | **Connective tissue autoimmune diseases** | 111 (25.65) | 38,039 (18.04) |  | 1.36 (1.12-1.65) | 1.36 (1.12-1.65) |
|  | Systemic lupus erythematosus | 58 (29.85) | 38,092 (18.04) |  | 1.54 (1.18-2.01) | 1.54 (1.18-2.01) |
|  | Sjögren’s disease | 32 (37.70) | 38,118 (18.05) |  | 1.93 (1.34-2.80) | 1.94 (1.34-2.80) |
|  | **Skin autoimmune diseases** | 240 (17.09) | 37,910 (18.06) |  | 0.95 (0.83-1.09) | 0.95 (0.83-1.09) |
|  | Psoriasis vulgaris | 206 (16.95) | 37,944 (18.06) |  | 0.95 (0.82-1.09) | 0.94 (0.82-1.09) |
|  | Vitiligo | 28 (18.24) | 38,122 (18.05) |  | 1.02 (0.69-1.51) | 1.02 (0.69-1.51) |
|  | **Blood autoimmune diseases** | 38 (19.06) | 38,112 (18.05) |  | 1.05 (0.75-1.46) | 1.05 (0.75-1.46) |
|  | Idiopathic thrombocytopenic purpura | 34 (21.21) | 38,116 (18.05) |  | 1.15 (0.81-1.64) | 1.15 (0.81-1.64) |
|  | **Nervous system autoimmune diseases** | 90 (20.12) | 38,060 (18.05) |  | 1.17 (0.95-1.44) | 1.17 (0.95-1.44) |
|  | Multiple sclerosis | 55 (17.47) | 38,095 (18.06) |  | 1.00 (0.76-1.31) | 0.99 (0.76-1.31) |
|  | **Gastrointestinal autoimmune diseases** | 532 (20.03) | 37,618 (18.03) |  | 1.12 (1.03-1.23) | 1.12 (1.03-1.23) |
|  | Crohn disease | 159 (20.26) | 37,991 (18.05) |  | 1.13 (0.97-1.33) | 1.13 (0.96-1.33) |
|  | Ulcerative colitis | 206 (19.61) | 37,944 (18.05) |  | 1.09 (0.95-1.25) | 1.09 (0.95-1.26) |
|  | Celiac disease | 213 (20.07) | 37,937 (18.04) |  | 1.14 (1.00-1.31) | 1.14 (1.00-1.31) |
|  | **Other autoimmune diseases** | 107 (20.99) | 38,043 (18.05) |  | 1.16 (0.95-1.40) | 1.15 (0.95-1.40) |
|  | Sarcoidosis | 27 (19.44) | 38,123 (18.05) |  | 1.11 (0.76-1.64) | 1.11 (0.76-1.64) |
|  | IgA nephropathy | 74 (22.02) | 38,076 (18.05) |  | 1.20 (0.96-1.51) | 1.20 (0.96-1.51) |
| **Vascular diseases** | **Any AID** | 325 (2.68) | 4588 (2.27) |  | 1.17 (1.04-1.33) | 1.17 (1.03-1.32) |
|  | **Endocrine autoimmune diseases** | 168 (2.95) | 4745 (2.28) |  | 1.27 (1.07-1.51) | 1.27 (1.07-1.51) |
|  | Type 1 Diabetes | 72 (5.11) | 4841 (2.28) |  | 1.73 (1.24-2.42) | 1.73 (1.23-2.41) |
|  | Autoimmune thyroiditis | 102 (2.29) | 4811 (2.30) |  | 1.12 (0.91-1.37) | 1.12 (0.91-1.38) |
|  | **Inflammatory arthritis** | 29 (2.68) | 4884 (2.30) |  | 1.21 (0.83-1.77) | 1.21 (0.83-1.76) |
|  | Rheumatoid arthritis | 22 (3.10) | 4891 (2.29) |  | 1.35 (0.87-2.10) | 1.35 (0.87-2.10) |
|  | **Skin autoimmune diseases** | 32 (2.26) | 4881 (2.30) |  | 1.00 (0.69-1.45) | 0.98 (0.68-1.43) |
|  | Psoriasis vulgaris | 26 (2.12) | 4887 (2.30) |  | 0.91 (0.60-1.38) | 0.88 (0.58-1.34) |
|  | **Gastrointestinal autoimmune diseases** | 72 (2.68) | 4841 (2.29) |  | 1.19 (0.93-1.52) | 1.19 (0.93-1.51) |
|  | Crohn disease | 24 (3.02) | 4889 (2.29) |  | 1.37 (0.91-2.06) | 1.35 (0.90-2.04) |
|  | Ulcerative colitis | 32 (3.01) | 4881 (2.29) |  | 1.29 (0.89-1.87) | 1.30 (0.90-1.89) |
| **Arrhythmias** | **Any AID** | 540 (4.46) | 7770 (3.86) |  | 1.18 (1.07-1.30) | 1.18 (1.07-1.29) |
|  | **Endocrine autoimmune diseases** | 272 (4.78) | 8038 (3.87) |  | 1.32 (1.15-1.51) | 1.32 (1.15-1.51) |
|  | Type 1 Diabetes | 79 (5.61) | 8231 (3.88) |  | 1.37 (1.01-1.86) | 1.37 (1.01-1.86) |
|  | Autoimmune thyroiditis | 204 (4.58) | 8106 (3.88) |  | 1.32 (1.14-1.53) | 1.32 (1.14-1.53) |
|  | **Inflammatory arthritis** | 45 (4.16) | 8265 (3.89) |  | 1.09 (0.80-1.47) | 1.09 (0.80-1.47) |
|  | Rheumatoid arthritis | 37 (5.22) | 8273 (3.89) |  | 1.33 (0.95-1.86) | 1.33 (0.95-1.86) |
|  | **Vasculitis** | 22 (5.96) | 8288 (3.89) |  | 1.34 (0.85-2.10) | 1.34 (0.85-2.10) |
|  | **Connective tissue autoimmune diseases** | 34 (7.74) | 8276 (3.88) |  | 1.76 (1.23-2.52) | 1.76 (1.23-2.52) |
|  | **Skin autoimmune diseases** | 47 (3.32) | 8263 (3.89) |  | 0.86 (0.63-1.16) | 0.85 (0.63-1.16) |
|  | Psoriasis vulgaris | 41 (3.34) | 8269 (3.89) |  | 0.89 (0.65-1.23) | 0.89 (0.64-1.23) |
|  | **Gastrointestinal autoimmune diseases** | 111 (4.13) | 8199 (3.89) |  | 1.04 (0.85-1.26) | 1.04 (0.85-1.26) |
|  | Crohn disease | 29 (3.65) | 8281 (3.89) |  | 0.85 (0.57-1.25) | 0.85 (0.57-1.25) |
|  | Ulcerative colitis | 42 (3.95) | 8268 (3.89) |  | 0.98 (0.71-1.35) | 0.98 (0.71-1.35) |
|  | Celiac disease | 48 (4.47) | 8262 (3.89) |  | 1.19 (0.88-1.59) | 1.19 (0.88-1.59) |
|  | **Other autoimmune diseases** | 20 (3.88) | 8290 (3.89) |  | 0.94 (0.60-1.47) | 0.94 (0.60-1.47) |
| **Structural heart diseases** | **Any AID** | 272 (2.24) | 3318 (1.64) |  | 1.19 (1.04-1.36) | 1.19 (1.04-1.36) |
|  | **Endocrine autoimmune diseases** | 166 (2.92) | 3424 (1.64) |  | 1.37 (1.15-1.64) | 1.37 (1.15-1.64) |
|  | Type 1 Diabetes | 88 (6.27) | 3502 (1.65) |  | 2.31 (1.67-3.19) | 2.30 (1.67-3.18) |
|  | Autoimmune thyroiditis | 93 (2.09) | 3497 (1.67) |  | 1.17 (0.95-1.45) | 1.18 (0.95-1.46) |
|  | **Skin autoimmune diseases** | 27 (1.90) | 3563 (1.68) |  | 1.11 (0.75-1.65) | 1.10 (0.74-1.63) |
|  | Psoriasis vulgaris | 25 (2.04) | 3565 (1.68) |  | 1.19 (0.79-1.79) | 1.17 (0.78-1.76) |
|  | **Gastrointestinal autoimmune diseases** | 41 (1.52) | 3549 (1.68) |  | 0.97 (0.71-1.32) | 0.97 (0.71-1.32) |
|  | Celiac disease | 21 (1.95) | 3569 (1.68) |  | 1.21 (0.79-1.87) | 1.21 (0.79-1.86) |

AID: Autoimmune disease; CI: Confidence interval; CVD: cardiovascular diseases; HR: Hazard ratio; PY: Person-year.

Only autoimmune diseases with ≥ 20 cardiovascular disease events occurring in the exposed group were included in our analyses.

Models 1 were adjusted for offspring’s calendar year of birth, maternal country of birth, parity, age, education, income, and marital status in the year of childbirth, maternal cardiovascular and psychiatric diseases before childbirth, maternal pre-gestational hypertension, and pre-gestational diabetes, Models 2 were additionally adjusted for maternal smoking in early pregnancy.

Table S7. Hazard ratios and 95% confidence intervals for cardiovascular diseases in offspring according to maternal autoimmune diseases during pregnancy in individuals who had data on maternal body mass index in early pregnancy (n= 1,321,186)

| Outcome | Exposure | Events (per 10,000 PYs) | |  | HR (95% CI) | |
| --- | --- | --- | --- | --- | --- | --- |
|  |  | Exposed | Non-exposed |  | Model 1 | Model 2 |
| **Any CVD** | **Any AID** | 2380 (20.89) | 33,484 (17.82) |  | 1.18 (1.13-1.24) | 1.18 (1.13-1.24) |
|  | **Endocrine autoimmune diseases** | 1187 (22.10) | 34,677 (17.88) |  | 1.25 (1.17-1.34) | 1.25 (1.17-1.34) |
|  | Type 1 Diabetes | 334 (25.34) | 35,530 (17.95) |  | 1.32 (1.14-1.53) | 1.32 (1.14-1.53) |
|  | Autoimmune thyroiditis | 903 (21.43) | 34,961 (17.92) |  | 1.25 (1.16-1.34) | 1.25 (1.16-1.34) |
|  | **Inflammatory arthritis** | 211 (20.93) | 35,653 (17.98) |  | 1.19 (1.03-1.36) | 1.19 (1.03-1.36) |
|  | Reactive arthritis | 41 (16.62) | 35,823 (18.00) |  | 1.01 (0.75-1.38) | 1.01 (0.75-1.38) |
|  | Rheumatoid arthritis | 147 (22.26) | 35,717 (17.98) |  | 1.22 (1.03-1.44) | 1.22 (1.03-1.44) |
|  | Ankylosing spondylitis | 34 (23.47) | 35,830 (17.99) |  | 1.38 (0.98-1.94) | 1.38 (0.98-1.94) |
|  | **Vasculitis** | 87 (25.45) | 35,777 (17.98) |  | 1.27 (1.02-1.60) | 1.27 (1.02-1.60) |
|  | Henoch-Schonlein purpura | 61 (23.62) | 35,803 (17.99) |  | 1.17 (0.89-1.54) | 1.17 (0.89-1.54) |
|  | **Connective tissue autoimmune diseases** | 105 (25.72) | 35,759 (17.98) |  | 1.37 (1.12-1.67) | 1.37 (1.12-1.67) |
|  | Systemic lupus erythematosus | 52 (28.21) | 35,812 (17.99) |  | 1.45 (1.09-1.93) | 1.45 (1.09-1.93) |
|  | Sjögren’s disease | 30 (37.64) | 35,834 (17.99) |  | 1.92 (1.31-2.82) | 1.92 (1.31-2.82) |
|  | **Skin autoimmune diseases** | 234 (17.5) | 35,630 (18.00) |  | 0.98 (0.85-1.12) | 0.98 (0.85-1.12) |
|  | Psoriasis vulgaris | 202 (17.47) | 35,662 (18.00) |  | 0.98 (0.85-1.13) | 0.98 (0.85-1.13) |
|  | Vitiligo | 26 (17.50) | 35,838 (18.00) |  | 0.97 (0.64-1.46) | 0.97 (0.64-1.46) |
|  | **Blood autoimmune diseases** | 37 (19.61) | 35,827 (17.99) |  | 1.09 (0.78-1.52) | 1.09 (0.78-1.52) |
|  | Idiopathic thrombocytopenic purpura | 33 (21.86) | 35,831 (17.99) |  | 1.19 (0.83-1.70) | 1.19 (0.83-1.70) |
|  | **Nervous system autoimmune diseases** | 87 (20.46) | 35,777 (17.99) |  | 1.19 (0.96-1.48) | 1.19 (0.96-1.48) |
|  | Multiple sclerosis | 54 (17.94) | 35,810 (18.00) |  | 1.03 (0.78-1.35) | 1.03 (0.78-1.35) |
|  | **Gastrointestinal autoimmune diseases** | 500 (19.74) | 35,364 (17.97) |  | 1.12 (1.02-1.23) | 1.12 (1.02-1.23) |
|  | Crohn disease | 148 (19.95) | 35,716 (17.99) |  | 1.13 (0.96-1.33) | 1.13 (0.96-1.33) |
|  | Ulcerative colitis | 197 (19.64) | 35,667 (17.99) |  | 1.10 (0.95-1.27) | 1.10 (0.95-1.27) |
|  | Celiac disease | 202 (19.86) | 35,662 (17.99) |  | 1.14 (0.99-1.31) | 1.14 (0.99-1.31) |
|  | **Other autoimmune diseases** | 98 (20.23) | 35,766 (17.99) |  | 1.14 (0.93-1.39) | 1.14 (0.93-1.39) |
|  | Sarcoidosis | 23 (17.32) | 35,841 (18.00) |  | 1.04 (0.69-1.56) | 1.04 (0.69-1.56) |
|  | IgA nephropathy | 69 (21.67) | 35,795 (17.99) |  | 1.20 (0.95-1.52) | 1.20 (0.95-1.52) |
| **Vascular diseases** | **Any AID** | 312 (2.70) | 4297 (2.26) |  | 1.20 (1.06-1.36) | 1.20 (1.05-1.35) |
|  | **Endocrine autoimmune diseases** | 162 (2.97) | 4447 (2.27) |  | 1.30 (1.09-1.56) | 1.29 (1.08-1.54) |
|  | Type 1 Diabetes | 69 (5.14) | 4540 (2.27) |  | 1.76 (1.25-2.46) | 1.83 (1.31-2.56) |
|  | Autoimmune thyroiditis | 98 (2.30) | 4511 (2.28) |  | 1.14 (0.92-1.41) | 1.11 (0.90-1.37) |
|  | **Inflammatory arthritis** | 28 (2.74) | 4581 (2.28) |  | 1.26 (0.85-1.85) | 1.26 (0.86-1.85) |
|  | Rheumatoid arthritis | 21 (3.14) | 4588 (2.28) |  | 1.39 (0.88-2.18) | 1.40 (0.89-2.19) |
|  | **Skin autoimmune diseases** | 32 (2.37) | 4577 (2.28) |  | 1.06 (0.73-1.54) | 1.04 (0.71-1.50) |
|  | Psoriasis vulgaris | 26 (2.23) | 4583 (2.29) |  | 0.96 (0.63-1.46) | 0.93 (0.61-1.42) |
|  | **Gastrointestinal autoimmune diseases** | 66 (2.57) | 4543 (2.28) |  | 1.15 (0.89-1.48) | 1.18 (0.91-1.51) |
|  | Crohn disease | 21 (2.79) | 4588 (2.28) |  | 1.27 (0.82-1.97) | 1.29 (0.83-1.99) |
|  | Ulcerative colitis | 30 (2.96) | 4579 (2.28) |  | 1.30 (0.89-1.91) | 1.32 (0.91-1.93) |
| **Arrhythmias** | **Any AID** | 512 (4.44) | 7279 (3.83) |  | 1.19 (1.08-1.31) | 1.19 (1.08-1.31) |
|  | **Endocrine autoimmune diseases** | 259 (4.76) | 7532 (3.84) |  | 1.33 (1.16-1.53) | 1.33 (1.16-1.53) |
|  | Type 1 Diabetes | 76 (5.66) | 7715 (3.86) |  | 1.43 (1.04-1.95) | 1.42 (1.04-1.95) |
|  | Autoimmune thyroiditis | 193 (4.53) | 7598 (3.85) |  | 1.32 (1.13-1.53) | 1.32 (1.13-1.53) |
|  | **Inflammatory arthritis** | 43 (4.21) | 7748 (3.87) |  | 1.11 (0.81-1.51) | 1.11 (0.81-1.51) |
|  | Rheumatoid arthritis | 36 (5.39) | 7755 (3.86) |  | 1.38 (0.98-1.94) | 1.38 (0.98-1.94) |
|  | **Vasculitis** | 21 (6.05) | 7770 (3.86) |  | 1.36 (0.85-2.15) | 1.36 (0.85-2.15) |
|  | **Connective tissue autoimmune diseases** | 30 (7.24) | 7761 (3.86) |  | 1.62 (1.10-2.38) | 1.62 (1.10-2.38) |
|  | **Skin autoimmune diseases** | 47 (3.48) | 7744 (3.87) |  | 0.91 (0.67-1.23) | 0.91 (0.67-1.23) |
|  | Psoriasis vulgaris | 41 (3.51) | 7750 (3.87) |  | 0.94 (0.68-1.30) | 0.94 (0.68-1.31) |
|  | **Gastrointestinal autoimmune diseases** | 104 (4.06) | 7687 (3.86) |  | 1.05 (0.86-1.28) | 1.05 (0.86-1.28) |
|  | Crohn disease | 27 (3.60) | 7764 (3.87) |  | 0.90 (0.61-1.34) | 0.90 (0.61-1.34) |
|  | Ulcerative colitis | 41 (4.04) | 7750 (3.87) |  | 1.03 (0.75-1.42) | 1.03 (0.75-1.42) |
|  | Celiac disease | 45 (4.37) | 7746 (3.86) |  | 1.16 (0.86-1.57) | 1.16 (0.86-1.57) |
| **Structural heart diseases** | **Any AID** | 264 (2.29) | 3129 (1.65) |  | 1.22 (1.07-1.40) | 1.22 (1.07-1.40) |
|  | **Endocrine autoimmune diseases** | 162 (2.98) | 3231 (1.65) |  | 1.42 (1.18-1.70) | 1.41 (1.18-1.69) |
|  | Type 1 Diabetes | 86 (6.43) | 3307 (1.65) |  | 2.43 (1.75-3.36) | 2.48 (1.79-3.42) |
|  | Autoimmune thyroiditis | 92 (2.16) | 3301 (1.67) |  | 1.22 (0.98-1.51) | 1.20 (0.97-1.49) |
|  | **Skin autoimmune diseases** | 25 (1.85) | 3368 (1.68) |  | 1.07 (0.71-1.62) | 1.06 (0.70-1.60) |
|  | Psoriasis vulgaris | 23 (1.97) | 3370 (1.68) |  | 1.14 (0.74-1.75) | 1.12 (0.73-1.72) |
|  | **Gastrointestinal autoimmune diseases** | 40 (1.56) | 3353 (1.68) |  | 0.98 (0.72-1.35) | 0.99 (0.73-1.36) |
|  | Celiac disease | 20 (1.94) | 3373 (1.68) |  | 1.20 (0.77-1.86) | 1.22 (0.78-1.89) |

AID: Autoimmune disease; CI: Confidence interval; CVD: cardiovascular diseases; HR: Hazard ratio; PY: Person-year.

Only autoimmune diseases with ≥ 20 cardiovascular disease events occurring in the exposed group were included in our analyses.

Models 1 were adjusted for offspring’s calendar year of birth, maternal country of birth, parity, age, education, income, and marital status in the year of childbirth, maternal cardiovascular and psychiatric diseases before childbirth, maternal pre-gestational hypertension, and pre-gestational diabetes, Models 2 were additionally adjusted for maternal body mass index in early pregnancy.

Table S8. Hazard ratios and 95% confidence intervals for cardiovascular diseases in offspring according to maternal autoimmune diseases during pregnancy in individuals who had data on maternal family history of CVD (n= 1,209,500)

| Outcome | Exposure | Events (per 10,000 PYs) | |  | HR (95% CI) | |
| --- | --- | --- | --- | --- | --- | --- |
|  |  | Exposed | Non-exposed |  | Model 1 | Model 2 |
| **Any CVD** | **Any AID** | 2374 (21.33) | 32,008 (18.22) |  | 1.19 (1.13-1.24) | 1.18 (1.13-1.24) |
|  | **Endocrine autoimmune diseases** | 1100 (22.47) | 33,282 (18.30) |  | 1.24 (1.16-1.33) | 1.24 (1.16-1.33) |
|  | Type 1 Diabetes | 308 (24.50) | 34,074 (18.37) |  | 1.21 (1.04-1.41) | 1.21 (1.04-1.42) |
|  | Autoimmune thyroiditis | 837 (22.06) | 33,545 (18.33) |  | 1.26 (1.17-1.35) | 1.26 (1.17-1.35) |
|  | **Inflammatory arthritis** | 219 (20.78) | 34,163 (18.39) |  | 1.16 (1.01-1.33) | 1.16 (1.01-1.33) |
|  | Reactive arthritis | 42 (16.51) | 34,340 (18.41) |  | 0.98 (0.72-1.33) | 0.98 (0.72-1.33) |
|  | Rheumatoid arthritis | 154 (22.13) | 34,228 (18.39) |  | 1.20 (1.02-1.42) | 1.20 (1.02-1.42) |
|  | Ankylosing spondylitis | 35 (23.43) | 34,347 (18.40) |  | 1.35 (0.97-1.89) | 1.35 (0.96-1.88) |
|  | **Vasculitis** | 95 (26.00) | 34,287 (18.39) |  | 1.30 (1.05-1.61) | 1.30 (1.05-1.61) |
|  | Henoch-Schonlein purpura | 65 (23.34) | 34,317 (18.40) |  | 1.16 (0.89-1.50) | 1.16 (0.89-1.50) |
|  | **Connective tissue autoimmune diseases** | 120 (28.88) | 34,262 (18.39) |  | 1.51 (1.26-1.82) | 1.51 (1.25-1.82) |
|  | Systemic lupus erythematosus | 65 (34.59) | 34,317 (18.39) |  | 1.77 (1.37-2.28) | 1.76 (1.37-2.27) |
|  | Sjögren’s disease | 33 (42.96) | 34,349 (18.40) |  | 2.15 (1.50-3.10) | 2.15 (1.50-3.10) |
|  | **Skin autoimmune diseases** | 238 (17.65) | 34,144 (18.41) |  | 0.98 (0.86-1.12) | 0.98 (0.86-1.12) |
|  | Psoriasis vulgaris | 206 (17.54) | 34,176 (18.41) |  | 0.98 (0.85-1.13) | 0.98 (0.85-1.13) |
|  | Vitiligo | 27 (19.48) | 34,355 (18.41) |  | 1.06 (0.71-1.58) | 1.06 (0.71-1.58) |
|  | **Blood autoimmune diseases** | 38 (20.14) | 34,344 (18.41) |  | 1.05 (0.75-1.47) | 1.05 (0.75-1.47) |
|  | Idiopathic thrombocytopenic purpura | 33 (22.04) | 34,349 (18.41) |  | 1.12 (0.78-1.62) | 1.12 (0.78-1.62) |
|  | **Nervous system autoimmune diseases** | 87 (20.12) | 34,295 (18.40) |  | 1.16 (0.94-1.43) | 1.16 (0.93-1.43) |
|  | Multiple sclerosis | 55 (18.02) | 34,327 (18.41) |  | 1.03 (0.78-1.34) | 1.03 (0.78-1.34) |
|  | **Gastrointestinal autoimmune diseases** | 535 (20.07) | 33,847 (18.38) |  | 1.11 (1.02-1.21) | 1.11 (1.02-1.21) |
|  | Crohn disease | 156 (19.94) | 34,226 (18.40) |  | 1.09 (0.93-1.28) | 1.09 (0.93-1.28) |
|  | Ulcerative colitis | 204 (19.50) | 34,178 (18.40) |  | 1.05 (0.91-1.22) | 1.05 (0.91-1.22) |
|  | Celiac disease | 224 (20.74) | 34,158 (18.39) |  | 1.17 (1.02-1.33) | 1.16 (1.02-1.33) |
|  | **Other autoimmune diseases** | 107 (21.91) | 34,275 (18.40) |  | 1.19 (0.98-1.44) | 1.19 (0.98-1.44) |
|  | Sarcoidosis | 28 (21.08) | 34,354 (18.41) |  | 1.19 (0.81-1.73) | 1.18 (0.81-1.73) |
|  | IgA nephropathy | 73 (22.69) | 34,309 (18.4) |  | 1.22 (0.97-1.54) | 1.22 (0.97-1.54) |
| **Vascular diseases** | **Any AID** | 302 (2.68) | 4076 (2.29) |  | 1.16 (1.02-1.32) | 1.16 (1.02-1.32) |
|  | **Endocrine autoimmune diseases** | 148 (2.98) | 4230 (2.30) |  | 1.25 (1.04-1.51) | 1.25 (1.04-1.51) |
|  | Type 1 Diabetes | 64 (5.00) | 4314 (2.30) |  | 1.59 (1.11-2.29) | 1.60 (1.11-2.29) |
|  | Autoimmune thyroiditis | 91 (2.37) | 4287 (2.31) |  | 1.15 (0.93-1.43) | 1.15 (0.92-1.43) |
|  | **Inflammatory arthritis** | 31 (2.90) | 4347 (2.31) |  | 1.32 (0.92-1.90) | 1.32 (0.91-1.90) |
|  | Rheumatoid arthritis | 24 (3.40) | 4354 (2.31) |  | 1.51 (0.99-2.29) | 1.50 (0.99-2.29) |
|  | **Skin autoimmune diseases** | 32 (2.35) | 4346 (2.32) |  | 1.07 (0.74-1.54) | 1.07 (0.74-1.54) |
|  | Psoriasis vulgaris | 26 (2.19) | 4352 (2.32) |  | 0.97 (0.64-1.46) | 0.97 (0.64-1.46) |
|  | **Gastrointestinal autoimmune diseases** | 66 (2.44) | 4312 (2.31) |  | 1.08 (0.84-1.39) | 1.08 (0.84-1.39) |
|  | Crohn disease | 21 (2.65) | 4357 (2.31) |  | 1.24 (0.81-1.90) | 1.24 (0.81-1.90) |
|  | Ulcerative colitis | 29 (2.74) | 4349 (2.31) |  | 1.14 (0.77-1.69) | 1.14 (0.77-1.69) |
|  | Celiac disease | 20 (1.83) | 4358 (2.32) |  | 0.79 (0.50-1.26) | 0.79 (0.50-1.25) |
| **Arrhythmias** | **Any AID** | 518 (4.6) | 7031 (3.96) |  | 1.20 (1.09-1.32) | 1.19 (1.09-1.32) |
|  | **Endocrine autoimmune diseases** | 239 (4.82) | 7310 (3.98) |  | 1.33 (1.15-1.53) | 1.32 (1.15-1.53) |
|  | Type 1 Diabetes | 61 (4.77) | 7488 (3.99) |  | 1.15 (0.81-1.63) | 1.15 (0.81-1.63) |
|  | Autoimmune thyroiditis | 187 (4.87) | 7362 (3.98) |  | 1.37 (1.18-1.59) | 1.37 (1.17-1.59) |
|  | **Inflammatory arthritis** | 43 (4.03) | 7506 (4.00) |  | 1.03 (0.76-1.41) | 1.03 (0.76-1.41) |
|  | Rheumatoid arthritis | 35 (4.97) | 7514 (3.99) |  | 1.25 (0.88-1.76) | 1.24 (0.88-1.76) |
|  | **Vasculitis** | 23 (6.20) | 7526 (3.99) |  | 1.38 (0.89-2.15) | 1.39 (0.89-2.15) |
|  | **Connective tissue autoimmune diseases** | 38 (8.99) | 7511 (3.99) |  | 1.96 (1.40-2.77) | 1.96 (1.39-2.76) |
|  | Systemic lupus erythematosus | 21 (10.96) | 7528 (3.99) |  | 2.21 (1.37-3.55) | 2.20 (1.37-3.55) |
|  | **Skin autoimmune diseases** | 47 (3.45) | 7502 (4.00) |  | 0.87 (0.64-1.19) | 0.87 (0.64-1.19) |
|  | Psoriasis vulgaris | 42 (3.54) | 7507 (4.00) |  | 0.93 (0.68-1.28) | 0.93 (0.67-1.28) |
|  | **Gastrointestinal autoimmune diseases** | 120 (4.45) | 7429 (3.99) |  | 1.09 (0.90-1.32) | 1.09 (0.90-1.32) |
|  | Crohn disease | 31 (3.91) | 7518 (4.00) |  | 0.87 (0.59-1.28) | 0.87 (0.59-1.28) |
|  | Ulcerative colitis | 45 (4.25) | 7504 (4.00) |  | 0.99 (0.73-1.36) | 0.99 (0.73-1.36) |
|  | Celiac disease | 54 (4.94) | 7495 (3.99) |  | 1.31 (0.99-1.73) | 1.31 (0.99-1.72) |
|  | **Other autoimmune diseases** | 21 (4.25) | 7528 (4.00) |  | 1.01 (0.65-1.57) | 1.01 (0.65-1.57) |
| **Structural heart diseases** | **Any AID** | 263 (2.33) | 2867 (1.61) |  | 1.24 (1.08-1.43) | 1.24 (1.08-1.42) |
|  | **Endocrine autoimmune diseases** | 150 (3.02) | 2980 (1.62) |  | 1.42 (1.17-1.72) | 1.42 (1.17-1.71) |
|  | Type 1 Diabetes | 81 (6.35) | 3049 (1.62) |  | 2.22 (1.56-3.17) | 2.23 (1.56-3.18) |
|  | Autoimmune thyroiditis | 83 (2.16) | 3047 (1.64) |  | 1.25 (1.00-1.56) | 1.24 (0.99-1.55) |
|  | **Skin autoimmune diseases** | 28 (2.06) | 3102 (1.65) |  | 1.23 (0.84-1.80) | 1.23 (0.84-1.79) |
|  | Psoriasis vulgaris | 26 (2.19) | 3104 (1.65) |  | 1.31 (0.88-1.94) | 1.30 (0.88-1.93) |
|  | **Gastrointestinal autoimmune diseases** | 43 (1.59) | 3087 (1.66) |  | 0.99 (0.73-1.33) | 0.99 (0.73-1.33) |
|  | Celiac disease | 23 (2.10) | 3107 (1.65) |  | 1.26 (0.83-1.90) | 1.26 (0.83-1.89) |

AID: Autoimmune disease; CI: Confidence interval; CVD: cardiovascular diseases; HR: Hazard ratio; PY: Person-year.

Only autoimmune diseases with ≥ 20 cardiovascular disease events occurring in the exposed group were included in our analyses.

Models 1 were adjusted for offspring’s calendar year of birth, maternal country of birth, parity, age, education, income, and marital status in the year of childbirth, maternal cardiovascular and psychiatric diseases before childbirth, maternal pre-gestational hypertension, and pre-gestational diabetes, Models 2 were additionally adjusted for maternal family history of cardiovascular diseases.

Table S9. Hazard ratios and 95% confidence intervals for cardiovascular diseases in offspring according to maternal autoimmune diseases during pregnancy in individuals who had data on preeclampsia, placental abruption, and gestational age at delivery

| Outcome | Exposure | Preeclampsia† | Placental abruption‡ | Gestational age at delivery (n= 1,455,033) | | | |
| --- | --- | --- | --- | --- | --- | --- | --- |
|  |  | HR (95% CI) | HR (95% CI) | Events (per 10,000 PYs) | | HR (95% CI)* | |
|  |  |  |  | Exposed | Non-exposed | Model 1 | Model 2 |
| **Any CVD** | **Any AID** | 1.19 (1.14-1.24) | 1.19 (1.14-1.24) | 2667 (21.30) | 37593 (18.02) | 1.19 (1.14-1.24) | 1.16 (1.11-1.21) |
|  | **Endocrine autoimmune diseases** | 1.25 (1.17-1.33) | 1.24 (1.17-1.32) | 1319 (22.42) | 38941 (18.09) | 1.25 (1.17-1.32) | 1.22 (1.14-1.29) |
|  | Type 1 Diabetes | 1.30 (1.13-1.49) | 1.30 (1.13-1.49) | 373 (25.35) | 39887 (18.16) | 1.30 (1.13-1.49) | 1.22 (1.06-1.40) |
|  | Autoimmune thyroiditis | 1.25 (1.17-1.33) | 1.25 (1.17-1.33) | 1002 (21.84) | 39258 (18.13) | 1.25 (1.17-1.33) | 1.23 (1.15-1.31) |
|  | **Inflammatory arthritis** | 1.17 (1.03-1.34) | 1.17 (1.03-1.34) | 234 (20.90) | 40026 (18.19) | 1.17 (1.03-1.34) | 1.14 (1.00-1.30) |
|  | Reactive arthritis | 0.99 (0.74-1.32) | 0.99 (0.74-1.32) | 45 (16.49) | 40215 (18.21) | 0.99 (0.74-1.32) | 0.97 (0.73-1.31) |
|  | Rheumatoid arthritis | 1.22 (1.04-1.43) | 1.22 (1.04-1.43) | 165 (22.44) | 40095 (18.19) | 1.22 (1.04-1.43) | 1.19 (1.01-1.39) |
|  | Ankylosing spondylitis | 1.31 (0.94-1.82) | 1.30 (0.94-1.82) | 36 (22.64) | 40224 (18.20) | 1.31 (0.94-1.82) | 1.26 (0.90-1.75) |
|  | **Vasculitis** | 1.29 (1.05-1.60) | 1.29 (1.05-1.59) | 99 (26.03) | 40161 (18.19) | 1.29 (1.05-1.59) | 1.28 (1.04-1.58) |
|  | Henoch-Schonlein purpura | 1.16 (0.90-1.51) | 1.16 (0.90-1.50) | 68 (23.69) | 40192 (18.20) | 1.16 (0.90-1.50) | 1.16 (0.90-1.50) |
|  | **Connective tissue autoimmune diseases** | 1.45 (1.21-1.74) | 1.45 (1.21-1.74) | 126 (27.66) | 40134 (18.19) | 1.45 (1.21-1.74) | 1.37 (1.14-1.64) |
|  | Systemic lupus erythematosus | 1.64 (1.28-2.11) | 1.64 (1.28-2.11) | 67 (32.33) | 40193 (18.19) | 1.65 (1.28-2.12) | 1.51 (1.17-1.94) |
|  | Sjögren’s disease | 2.04 (1.43-2.90) | 2.04 (1.43-2.89) | 35 (39.77) | 40225 (18.20) | 2.04 (1.43-2.90) | 1.95 (1.37-2.78) |
|  | **Skin autoimmune diseases** | 0.98 (0.86-1.12) | 0.98 (0.86-1.12) | 258 (17.66) | 40002 (18.21) | 0.98 (0.86-1.12) | 0.97 (0.86-1.11) |
|  | Psoriasis vulgaris | 0.97 (0.85-1.12) | 0.97 (0.85-1.12) | 221 (17.47) | 40039 (18.21) | 0.97 (0.85-1.12) | 0.97 (0.84-1.11) |
|  | Vitiligo | 1.08 (0.75-1.57) | 1.08 (0.75-1.57) | 31 (19.45) | 40229 (18.20) | 1.08 (0.75-1.57) | 1.07 (0.74-1.56) |
|  | **Blood autoimmune diseases** | 1.04 (0.75-1.43) | 1.04 (0.75-1.43) | 42 (19.87) | 40218 (18.20) | 1.04 (0.75-1.43) | 1.01 (0.73-1.39) |
|  | Idiopathic thrombocytopenic purpura | 1.12 (0.79-1.58) | 1.12 (0.79-1.58) | 37 (21.86) | 40223 (18.20) | 1.12 (0.79-1.58) | 1.08 (0.77-1.53) |
|  | **Nervous system autoimmune diseases** | 1.15 (0.93-1.41) | 1.15 (0.93-1.41) | 93 (19.88) | 40167 (18.20) | 1.15 (0.93-1.41) | 1.13 (0.92-1.39) |
|  | Acute disseminated encephalomyelitis | 2.13 (1.38-3.31) | 2.12 (1.37-3.29) | 20 (35.08) | 40240 (18.20) | 2.13 (1.37-3.30) | 2.10 (1.36-3.26) |
|  | Multiple sclerosis | 0.98 (0.75-1.28) | 0.98 (0.75-1.28) | 57 (17.30) | 40203 (18.21) | 0.98 (0.75-1.28) | 0.96 (0.74-1.26) |
|  | **Gastrointestinal autoimmune diseases** | 1.12 (1.03-1.22) | 1.12 (1.03-1.22) | 564 (20.27) | 39696 (18.18) | 1.12 (1.03-1.22) | 1.10 (1.01-1.20) |
|  | Crohn disease | 1.11 (0.95-1.30) | 1.11 (0.95-1.30) | 167 (20.28) | 40093 (18.20) | 1.11 (0.95-1.30) | 1.08 (0.92-1.26) |
|  | Ulcerative colitis | 1.07 (0.94-1.23) | 1.07 (0.94-1.23) | 219 (19.84) | 40041 (18.20) | 1.08 (0.94-1.23) | 1.05 (0.91-1.20) |
|  | Celiac disease | 1.16 (1.02-1.33) | 1.16 (1.02-1.33) | 229 (20.64) | 40031 (18.19) | 1.17 (1.02-1.33) | 1.16 (1.02-1.33) |
|  | **Other autoimmune diseases** | 1.17 (0.97-1.41) | 1.16 (0.97-1.40) | 114 (21.37) | 40146 (18.20) | 1.17 (0.97-1.40) | 1.13 (0.94-1.36) |
|  | Sarcoidosis | 1.16 (0.81-1.67) | 1.16 (0.81-1.67) | 30 (20.49) | 40230 (18.20) | 1.16 (0.81-1.67) | 1.15 (0.80-1.66) |
|  | IgA nephropathy | 1.21 (0.97-1.52) | 1.20 (0.96-1.50) | 78 (22.25) | 40182 (18.20) | 1.20 (0.96-1.51) | 1.16 (0.92-1.45) |
| **Vascular diseases** | **Any AID** | 1.19 (1.05-1.34) | 1.18 (1.04-1.33) | 347 (2.73) | 4827 (2.29) | 1.18 (1.05-1.33) | 1.14 (1.01-1.28) |
|  | **Endocrine autoimmune diseases** | 1.30 (1.10-1.54) | 1.29 (1.09-1.53) | 182 (3.05) | 4992 (2.29) | 1.29 (1.09-1.53) | 1.23 (1.04-1.46) |
|  | Type 1 Diabetes | 1.74 (1.26-2.40) | 1.70 (1.23-2.35) | 77 (5.14) | 5097 (2.29) | 1.71 (1.24-2.36) | 1.55 (1.12-2.14) |
|  | Autoimmune thyroiditis | 1.18 (0.97-1.43) | 1.16 (0.96-1.42) | 113 (2.43) | 5061 (2.31) | 1.16 (0.96-1.42) | 1.13 (0.93-1.38) |
|  | **Inflammatory arthritis** | 1.24 (0.86-1.78) | 1.23 (0.85-1.77) | 31 (2.73) | 5143 (2.31) | 1.23 (0.85-1.77) | 1.18 (0.82-1.70) |
|  | Rheumatoid arthritis | 1.42 (0.93-2.15) | 1.40 (0.92-2.14) | 24 (3.22) | 5150 (2.31) | 1.41 (0.92-2.14) | 1.34 (0.88-2.04) |
|  | **Skin autoimmune diseases** | 1.02 (0.71-1.46) | 1.02 (0.71-1.46) | 34 (2.30) | 5140 (2.31) | 1.02 (0.71-1.46) | 1.01 (0.70-1.44) |
|  | Psoriasis vulgaris | 0.94 (0.63-1.40) | 0.93 (0.62-1.39) | 28 (2.19) | 5146 (2.31) | 0.93 (0.63-1.40) | 0.92 (0.62-1.38) |
|  | **Gastrointestinal autoimmune diseases** | 1.14 (0.90-1.45) | 1.14 (0.90-1.45) | 74 (2.63) | 5100 (2.31) | 1.14 (0.90-1.46) | 1.11 (0.87-1.41) |
|  | Crohn disease | 1.29 (0.86-1.95) | 1.29 (0.85-1.94) | 24 (2.88) | 5150 (2.31) | 1.29 (0.86-1.95) | 1.23 (0.82-1.85) |
|  | Ulcerative colitis | 1.22 (0.84-1.77) | 1.22 (0.84-1.77) | 33 (2.95) | 5141 (2.31) | 1.22 (0.84-1.77) | 1.17 (0.81-1.69) |
|  | Celiac disease | 0.82 (0.52-1.28) | 0.82 (0.52-1.28) | 21 (1.87) | 5153 (2.31) | 0.82 (0.52-1.28) | 0.81 (0.52-1.27) |
| **Arrhythmias** | **Any AID** | 1.19 (1.08-1.30) | 1.18 (1.08-1.30) | 573 (4.52) | 8167 (3.87) | 1.19 (1.08-1.30) | 1.16 (1.06-1.27) |
|  | **Endocrine autoimmune diseases** | 1.30 (1.13-1.48) | 1.29 (1.13-1.48) | 282 (4.73) | 8458 (3.89) | 1.30 (1.13-1.48) | 1.26 (1.10-1.44) |
|  | Type 1 Diabetes | 1.35 (1.00-1.82) | 1.35 (1.00-1.83) | 82 (5.47) | 8658 (3.90) | 1.35 (1.00-1.83) | 1.26 (0.93-1.70) |
|  | Autoimmune thyroiditis | 1.29 (1.12-1.49) | 1.29 (1.12-1.49) | 211 (4.55) | 8529 (3.90) | 1.29 (1.12-1.49) | 1.27 (1.10-1.46) |
|  | **Inflammatory arthritis** | 1.08 (0.80-1.45) | 1.08 (0.80-1.45) | 47 (4.15) | 8693 (3.91) | 1.08 (0.80-1.45) | 1.05 (0.78-1.41) |
|  | Rheumatoid arthritis | 1.29 (0.93-1.80) | 1.29 (0.93-1.80) | 38 (5.10) | 8702 (3.91) | 1.29 (0.93-1.80) | 1.25 (0.90-1.74) |
|  | **Vasculitis** | 1.40 (0.92-2.16) | 1.40 (0.91-2.15) | 24 (6.21) | 8716 (3.91) | 1.40 (0.91-2.15) | 1.39 (0.90-2.13) |
|  | **Connective tissue autoimmune diseases** | 1.88 (1.34-2.63) | 1.88 (1.34-2.63) | 39 (8.43) | 8701 (3.90) | 1.88 (1.34-2.64) | 1.76 (1.25-2.46) |
|  | Systemic lupus erythematosus | 2.04 (1.27-3.29) | 2.04 (1.27-3.29) | 21 (9.94) | 8719 (3.90) | 2.05 (1.27-3.30) | 1.84 (1.14-2.96) |
|  | **Skin autoimmune diseases** | 0.83 (0.61-1.13) | 0.83 (0.61-1.13) | 48 (3.25) | 8692 (3.91) | 0.83 (0.62-1.13) | 0.83 (0.61-1.12) |
|  | Psoriasis vulgaris | 0.87 (0.63-1.20) | 0.87 (0.63-1.20) | 42 (3.29) | 8698 (3.91) | 0.87 (0.63-1.20) | 0.86 (0.63-1.19) |
|  | **Gastrointestinal autoimmune diseases** | 1.08 (0.89-1.30) | 1.08 (0.89-1.30) | 123 (4.37) | 8617 (3.90) | 1.08 (0.90-1.30) | 1.06 (0.88-1.28) |
|  | Crohn disease | 0.87 (0.59-1.26) | 0.86 (0.59-1.26) | 32 (3.84) | 8708 (3.91) | 0.87 (0.59-1.26) | 0.84 (0.57-1.22) |
|  | Ulcerative colitis | 0.97 (0.71-1.33) | 0.97 (0.71-1.33) | 46 (4.12) | 8694 (3.91) | 0.97 (0.71-1.33) | 0.94 (0.69-1.29) |
|  | Celiac disease | 1.30 (0.99-1.71) | 1.30 (0.99-1.71) | 55 (4.90) | 8685 (3.90) | 1.30 (0.99-1.71) | 1.30 (0.99-1.70) |
|  | **Other autoimmune diseases** | 0.99 (0.64-1.52) | 0.98 (0.64-1.51) | 22 (4.07) | 8718 (3.91) | 0.99 (0.64-1.52) | 0.95 (0.62-1.46) |
| **Structural heart diseases** | **Any AID** | 1.23 (1.08-1.40) | 1.23 (1.08-1.40) | 302 (2.38) | 3513 (1.66) | 1.23 (1.08-1.40) | 1.17 (1.03-1.33) |
|  | **Endocrine autoimmune diseases** | 1.42 (1.20-1.69) | 1.42 (1.19-1.68) | 184 (3.09) | 3631 (1.67) | 1.42 (1.20-1.68) | 1.33 (1.12-1.58) |
|  | Type 1 Diabetes | 2.38 (1.75-3.26) | 2.37 (1.73-3.24) | 96 (6.43) | 3719 (1.67) | 2.38 (1.74-3.25) | 2.10 (1.54-2.86) |
|  | Autoimmune thyroiditis | 1.24 (1.01-1.52) | 1.24 (1.01-1.51) | 106 (2.28) | 3709 (1.69) | 1.24 (1.01-1.52) | 1.19 (0.97-1.46) |
|  | **Inflammatory arthritis** | 1.04 (0.66-1.63) | 1.03 (0.66-1.62) | 20 (1.76) | 3795 (1.70) | 1.03 (0.66-1.62) | 0.98 (0.62-1.53) |
|  | **Skin autoimmune diseases** | 1.12 (0.77-1.64) | 1.12 (0.77-1.64) | 29 (1.96) | 3786 (1.70) | 1.12 (0.77-1.64) | 1.10 (0.76-1.61) |
|  | Psoriasis vulgaris | 1.20 (0.81-1.78) | 1.20 (0.81-1.78) | 27 (2.11) | 3788 (1.70) | 1.20 (0.81-1.78) | 1.18 (0.80-1.75) |
|  | **Gastrointestinal autoimmune diseases** | 1.01 (0.76-1.35) | 1.01 (0.76-1.35) | 46 (1.63) | 3769 (1.71) | 1.01 (0.76-1.36) | 0.98 (0.73-1.31) |
|  | Celiac disease | 1.34 (0.91-1.99) | 1.35 (0.91-2.00) | 25 (2.22) | 3790 (1.70) | 1.35 (0.91-2.00) | 1.33 (0.90-1.97) |

AID: Autoimmune disease; CI: Confidence interval; CVD: cardiovascular diseases; HR: Hazard ratio; PY: Person-year.

Only autoimmune diseases with ≥ 20 cardiovascular disease events occurring in the exposed group were included in our analyses.

†: There is no missing data on preeclampsia. The analyses were performed in the whole cohort and were adjusted for offspring’s calendar year of birth, maternal country of birth, parity, age, education, income, and marital status in the year of childbirth, maternal cardiovascular and psychiatric diseases before childbirth, and pre-gestational diabetes, and preeclampsia.

‡: There is no missing data on placental abruption. The analyses were performed in the whole cohort and were adjusted for offspring’s calendar year of birth, maternal country of birth, parity, age, education, income, and marital status in the year of childbirth, maternal cardiovascular and psychiatric diseases before childbirth, maternal pre-gestational hypertension, and pre-gestational diabetes, and placental abruption.

*: Models 1 were adjusted for offspring’s calendar year of birth, maternal country of birth, parity, age, education, income, and marital status in the year of childbirth, maternal cardiovascular and psychiatric diseases before childbirth, maternal pre-gestational hypertension, and pre-gestational diabetes, Models 2 were additionally adjusted for gestational age at delivery.

Table S10. Hazard ratios and 95% confidence intervals for cardiovascular diseases according to maternal autoimmune diseases in offspring without congenital heart disease

| Outcome | Exposure | Events (per 10,000 PYs) | | HR (95% CI) | | |
| --- | --- | --- | --- | --- | --- | --- |
|  |  | Exposed | Non-exposed | Model 1 | Model 2 | Model 3 |
| **Any CVD** | **Any AID** | 2253 (18.48) | 32,780 (16.03) | 1.21 (1.16-1.26) | 1.22 (1.16-1.27) | 1.19 (1.14-1.25) |
|  | Endocrine autoimmune diseases | 1084 (18.98) | 33,949 (16.09) | 1.26 (1.19-1.34) | 1.27 (1.20-1.36) | 1.25 (1.17-1.34) |
|  | Inflammatory arthritis | 207 (18.92) | 34,826 (16.15) | 1.22 (1.06-1.39) | 1.24 (1.08-1.43) | 1.21 (1.05-1.40) |
|  | Vasculitis | 84 (22.62) | 34,949 (16.16) | 1.43 (1.15-1.77) | 1.32 (1.05-1.66) | 1.27 (1.01-1.60) |
|  | Connective tissue autoimmune diseases | 98 (22.21) | 34,935 (16.16) | 1.40 (1.15-1.70) | 1.41 (1.15-1.73) | 1.33 (1.09-1.63) |
|  | Skin autoimmune diseases | 219 (15.38) | 34,814 (16.17) | 1.01 (0.89-1.16) | 1.00 (0.87-1.15) | 0.99 (0.86-1.14) |
|  | Blood autoimmune diseases | 38 (18.37) | 34,995 (16.17) | 1.17 (0.85-1.61) | 1.13 (0.81-1.58) | 1.10 (0.79-1.55) |
|  | Nervous system autoimmune diseases | 83 (18.13) | 34,950 (16.16) | 1.16 (0.93-1.44) | 1.20 (0.97-1.49) | 1.19 (0.96-1.48) |
|  | Gastrointestinal autoimmune diseases | 492 (18.11) | 34,541 (16.14) | 1.16 (1.06-1.27) | 1.15 (1.05-1.26) | 1.13 (1.03-1.24) |
|  | Other autoimmune diseases | 101 (19.46) | 34,932 (16.16) | 1.21 (1.00-1.47) | 1.26 (1.04-1.54) | 1.20 (0.98-1.46) |
| **Vascular diseases** | **Any AID** | 284 (2.30) | 4094 (1.98) | 1.32 (1.17-1.48) | 1.29 (1.14-1.47) | 1.17 (1.03-1.34) |
|  | Endocrine autoimmune diseases | 148 (2.56) | 4230 (1.98) | 1.52 (1.29-1.79) | 1.55 (1.30-1.83) | 1.31 (1.09-1.58) |
|  | Inflammatory arthritis | 23 (2.08) | 4355 (2.00) | 1.14 (0.76-1.72) | 1.15 (0.76-1.75) | 1.11 (0.73-1.69) |
|  | Skin autoimmune diseases | 28 (1.95) | 4350 (2.00) | 1.15 (0.79-1.66) | 1.05 (0.71-1.56) | 1.03 (0.69-1.52) |
|  | Gastrointestinal autoimmune diseases | 62 (2.26) | 4316 (2.00) | 1.23 (0.96-1.58) | 1.17 (0.90-1.52) | 1.14 (0.88-1.49) |
| **Arrhythmias** | **Any AID** | 463 (3.76) | 7061 (3.42) | 1.20 (1.09-1.32) | 1.18 (1.07-1.30) | 1.16 (1.05-1.28) |
|  | Endocrine autoimmune diseases | 220 (3.81) | 7304 (3.43) | 1.25 (1.09-1.43) | 1.27 (1.10-1.46) | 1.28 (1.10-1.48) |
|  | Inflammatory arthritis | 38 (3.43) | 7486 (3.44) | 1.07 (0.78-1.47) | 1.10 (0.80-1.52) | 1.07 (0.77-1.47) |
|  | Vasculitis | 21 (5.58) | 7503 (3.44) | 1.69 (1.10-2.59) | 1.50 (0.94-2.38) | 1.41 (0.89-2.24) |
|  | Connective tissue autoimmune diseases | 22 (4.92) | 7502 (3.44) | 1.47 (0.97-2.23) | 1.41 (0.91-2.19) | 1.30 (0.84-2.02) |
|  | Skin autoimmune diseases | 42 (2.92) | 7482 (3.44) | 0.95 (0.70-1.29) | 0.92 (0.67-1.26) | 0.90 (0.65-1.24) |
|  | Gastrointestinal autoimmune diseases | 108 (3.93) | 7416 (3.43) | 1.22 (1.01-1.47) | 1.13 (0.92-1.38) | 1.10 (0.90-1.34) |
| **Structural heart diseases** | **Any AID** | 117 (0.95) | 1382 (0.67) | 1.51 (1.25-1.83) | 1.58 (1.30-1.92) | 1.32 (1.08-1.62) |
|  | Endocrine autoimmune diseases | 70 (1.21) | 1429 (0.67) | 1.96 (1.54-2.50) | 2.00 (1.56-2.56) | 1.48 (1.12-1.95) |

AID: Autoimmune disease; CI: Confidence interval; CVD: cardiovascular diseases; HR: Hazard ratio; PY: Person-year.

Only autoimmune diseases with ≥ 20 cardiovascular disease events occurring in the exposed group were included in our analyses.

Models 1 were adjusted for age at follow-up; Models 2 were additionally adjusted for offspring’s calendar year of birth, maternal country of birth, parity, education, income, and marital status in the year of childbirth; Models 3 were adjusted for factors in model 2 and for maternal cardiovascular and psychiatric diseases before childbirth, maternal pre-gestational hypertension, and pre-gestational diabetes.

Table S11. Hazard ratios and 95% confidence intervals for cardiovascular diseases according to maternal autoimmune diseases in offspring without major malformations

| Outcome | Exposure | Events (per 10,000 PYs) | | HR (95% CI) | | |
| --- | --- | --- | --- | --- | --- | --- |
|  |  | Exposed | Non-exposed | Model 1 | Model 2 | Model 3 |
| **Any CVD** | **Any AID** | 2305 (19.89) | 33,129 (17.0) | 1.06 (1.02-1.10) | 1.23 (1.17-1.28) | 1.19 (1.14-1.25) |
|  | Endocrine autoimmune diseases | 1123 (20.73) | 34,311 (17.06) | 1.06 (1.00-1.13) | 1.29 (1.21-1.38) | 1.25 (1.17-1.33) |
|  | Inflammatory arthritis | 207 (19.86) | 35,227 (17.15) | 1.08 (0.94-1.24) | 1.21 (1.05-1.39) | 1.18 (1.02-1.36) |
|  | Vasculitis | 86 (24.43) | 35,348 (17.15) | 1.37 (1.11-1.70) | 1.35 (1.08-1.69) | 1.30 (1.03-1.62) |
|  | Connective tissue autoimmune diseases | 104 (24.69) | 35,330 (17.14) | 1.40 (1.15-1.70) | 1.48 (1.21-1.80) | 1.40 (1.15-1.70) |
|  | Skin autoimmune diseases | 221 (16.30) | 35,213 (17.17) | 0.84 (0.74-0.96) | 0.98 (0.85-1.12) | 0.96 (0.84-1.11) |
|  | Blood autoimmune diseases | 33 (16.77) | 35,401 (17.16) | 0.92 (0.65-1.29) | 1.02 (0.72-1.45) | 0.99 (0.70-1.41) |
|  | Nervous system autoimmune diseases | 84 (19.38) | 35,350 (17.16) | 1.07 (0.86-1.32) | 1.21 (0.97-1.50) | 1.20 (0.96-1.49) |
|  | Gastrointestinal autoimmune diseases | 508 (19.62) | 34,926 (17.13) | 1.07 (0.98-1.17) | 1.18 (1.07-1.29) | 1.15 (1.05-1.26) |
|  | Other autoimmune diseases | 99 (20.01) | 35,335 (17.15) | 1.15 (0.94-1.40) | 1.22 (1.00-1.49) | 1.15 (0.95-1.41) |
| **Vascular diseases** | **Any AID** | 296 (2.52) | 4200 (2.13) | 1.07 (0.95-1.21) | 1.31 (1.16-1.48) | 1.17 (1.03-1.34) |
|  | Endocrine autoimmune diseases | 154 (2.81) | 4342 (2.14) | 1.15 (0.98-1.35) | 1.54 (1.31-1.82) | 1.28 (1.06-1.53) |
|  | Inflammatory arthritis | 27 (2.56) | 4469 (2.15) | 1.11 (0.76-1.62) | 1.27 (0.86-1.88) | 1.22 (0.82-1.81) |
|  | Skin autoimmune diseases | 31 (2.26) | 4465 (2.15) | 0.94 (0.66-1.33) | 1.14 (0.78-1.65) | 1.11 (0.76-1.61) |
|  | Gastrointestinal autoimmune diseases | 65 (2.48) | 4431 (2.15) | 1.08 (0.84-1.37) | 1.21 (0.94-1.56) | 1.18 (0.92-1.53) |
| **Arrhythmias** | **Any AID** | 478 (4.08) | 7229 (3.67) | 1.01 (0.92-1.10) | 1.18 (1.07-1.30) | 1.14 (1.04-1.26) |
|  | Endocrine autoimmune diseases | 225 (4.10) | 7482 (3.68) | 0.98 (0.86-1.12) | 1.25 (1.09-1.43) | 1.22 (1.06-1.42) |
|  | Inflammatory arthritis | 41 (3.89) | 7666 (3.69) | 0.98 (0.72-1.33) | 1.12 (0.82-1.54) | 1.09 (0.79-1.49) |
|  | Vasculitis | 21 (5.88) | 7686 (3.69) | 1.54 (1.00-2.36) | 1.54 (0.98-2.42) | 1.46 (0.93-2.28) |
|  | Connective tissue autoimmune diseases | 32 (7.49) | 7675 (3.69) | 1.97 (1.40-2.79) | 2.06 (1.44-2.95) | 1.90 (1.32-2.71) |
|  | Skin autoimmune diseases | 40 (2.92) | 7667 (3.70) | 0.70 (0.52-0.96) | 0.79 (0.57-1.11) | 0.77 (0.55-1.08) |
|  | Gastrointestinal autoimmune diseases | 109 (4.16) | 7598 (3.69) | 1.05 (0.87-1.27) | 1.10 (0.90-1.35) | 1.07 (0.88-1.31) |
| **Structural heart diseases** | **Any AID** | 225 (1.92) | 2687 (1.36) | 1.28 (1.11-1.46) | 1.45 (1.26-1.67) | 1.23 (1.06-1.43) |
|  | Endocrine autoimmune diseases | 131 (2.39) | 2781 (1.37) | 1.53 (1.29-1.83) | 1.79 (1.49-2.14) | 1.35 (1.11-1.66) |
|  | Skin autoimmune diseases | 21 (1.53) | 2891 (1.39) | 0.98 (0.64-1.51) | 1.07 (0.68-1.68) | 1.04 (0.66-1.63) |
|  | Gastrointestinal autoimmune diseases | 39 (1.49) | 2873 (1.39) | 1.00 (0.73-1.37) | 1.17 (0.85-1.60) | 1.13 (0.82-1.55) |

AID: Autoimmune disease; CI: Confidence interval; CVD: cardiovascular diseases; HR: Hazard ratio; PY: Person-year.

Only autoimmune diseases with ≥ 20 cardiovascular disease events occurring in the exposed group were included in our analyses.

Models 1 were adjusted for age at follow-up; Models 2 were additionally adjusted for offspring’s calendar year of birth, maternal country of birth, parity, education, income, and marital status in the year of childbirth; Models 3 were adjusted for factors in model 2 and for maternal cardiovascular and psychiatric diseases before childbirth, maternal pre-gestational hypertension, and pre-gestational diabetes.

Table S12. Hazard ratios and 95% confidence intervals for cardiovascular diseases according to maternal autoimmune diseases in offspring without autoimmune diseases

| Outcome | Exposure | Events (per 10,000 PYs) | | HR (95% CI) | | |
| --- | --- | --- | --- | --- | --- | --- |
|  |  | Exposed | Non-exposed | Model 1 | Model 2 | Model 3 |
| **Any CVD** | **Any AID** | 2368 (20.33) | 34,234 (17.18) | 1.07 (1.02-1.11) | 1.23 (1.18-1.28) | 1.19 (1.13-1.24) |
|  | Endocrine autoimmune diseases | 1177 (21.46) | 35,425 (17.25) | 1.09 (1.02-1.15) | 1.30 (1.23-1.39) | 1.25 (1.17-1.33) |
|  | Inflammatory arthritis | 201 (19.34) | 36,401 (17.35) | 1.03 (0.90-1.19) | 1.16 (1.01-1.34) | 1.13 (0.98-1.31) |
|  | Vasculitis | 90 (25.05) | 36,512 (17.34) | 1.40 (1.14-1.72) | 1.36 (1.09-1.69) | 1.30 (1.04-1.62) |
|  | Connective tissue autoimmune diseases | 105 (24.97) | 36,497 (17.34) | 1.40 (1.15-1.69) | 1.44 (1.18-1.75) | 1.36 (1.11-1.66) |
|  | Skin autoimmune diseases | 232 (17.02) | 36,370 (17.36) | 0.87 (0.77-0.99) | 1.01 (0.88-1.15) | 0.99 (0.87-1.14) |
|  | Blood autoimmune diseases | 38 (19.25) | 36,564 (17.36) | 1.04 (0.76-1.43) | 1.10 (0.78-1.54) | 1.07 (0.76-1.50) |
|  | Nervous system autoimmune diseases | 85 (19.12) | 36,517 (17.35) | 1.04 (0.84-1.29) | 1.18 (0.95-1.46) | 1.16 (0.94-1.44) |
|  | Gastrointestinal autoimmune diseases | 487 (19.2) | 36,115 (17.33) | 1.03 (0.94-1.13) | 1.13 (1.03-1.24) | 1.11 (1.01-1.22) |
|  | Other autoimmune diseases | 96 (19.01) | 36,506 (17.35) | 1.08 (0.88-1.32) | 1.14 (0.93-1.40) | 1.08 (0.88-1.33) |
| **Vascular diseases** | **Any AID** | 278 (2.36) | 4038 (2.0) | 1.06 (0.94-1.20) | 1.29 (1.13-1.46) | 1.15 (1.01-1.31) |
|  | Endocrine autoimmune diseases | 150 (2.70) | 4166 (2.0) | 1.18 (1.00-1.38) | 1.55 (1.30-1.83) | 1.27 (1.06-1.54) |
|  | Inflammatory arthritis | 27 (2.57) | 4289 (2.02) | 1.18 (0.81-1.72) | 1.36 (0.92-2.01) | 1.31 (0.88-1.94) |
|  | Skin autoimmune diseases | 28 (2.03) | 4288 (2.02) | 0.89 (0.62-1.29) | 1.07 (0.72-1.59) | 1.04 (0.70-1.55) |
|  | Gastrointestinal autoimmune diseases | 58 (2.26) | 4258 (2.02) | 1.04 (0.80-1.35) | 1.17 (0.89-1.53) | 1.14 (0.87-1.50) |
| **Arrhythmias** | **Any AID** | 518 (4.39) | 7549 (3.75) | 1.06 (0.97-1.16) | 1.23 (1.12-1.36) | 1.18 (1.08-1.31) |
|  | Endocrine autoimmune diseases | 255 (4.59) | 7812 (3.76) | 1.07 (0.94-1.21) | 1.35 (1.18-1.54) | 1.29 (1.13-1.49) |
|  | Inflammatory arthritis | 43 (4.09) | 8024 (3.78) | 1.00 (0.74-1.35) | 1.13 (0.83-1.54) | 1.09 (0.80-1.49) |
|  | Vasculitis | 24 (6.59) | 8043 (3.78) | 1.69 (1.13-2.52) | 1.63 (1.06-2.49) | 1.53 (1.00-2.35) |
|  | Connective tissue autoimmune diseases | 31 (7.26) | 8036 (3.78) | 1.87 (1.31-2.66) | 1.75 (1.19-2.57) | 1.61 (1.10-2.37) |
|  | Skin autoimmune diseases | 44 (3.20) | 8023 (3.79) | 0.75 (0.56-1.01) | 0.88 (0.64-1.20) | 0.86 (0.63-1.17) |
|  | Gastrointestinal autoimmune diseases | 104 (4.05) | 7963 (3.78) | 1.00 (0.82-1.21) | 1.04 (0.85-1.28) | 1.02 (0.83-1.25) |
|  | Other autoimmune diseases | 21 (4.11) | 8046 (3.79) | 1.07 (0.70-1.65) | 1.11 (0.71-1.72) | 1.03 (0.66-1.59) |
| **Structural heart diseases** | **Any AID** | 268 (2.27) | 3101 (1.54) | 1.33 (1.18-1.51) | 1.50 (1.32-1.71) | 1.27 (1.10-1.45) |
|  | Endocrine autoimmune diseases | 160 (2.88) | 3209 (1.54) | 1.63 (1.39-1.91) | 1.87 (1.59-2.21) | 1.41 (1.17-1.69) |
|  | Skin autoimmune diseases | 27 (1.96) | 3342 (1.58) | 1.11 (0.76-1.62) | 1.28 (0.87-1.88) | 1.24 (0.84-1.82) |
|  | Gastrointestinal autoimmune diseases | 37 (1.44) | 3332 (1.58) | 0.85 (0.61-1.17) | 0.99 (0.72-1.37) | 0.96 (0.69-1.32) |

AID: Autoimmune disease; CI: Confidence interval; CVD: cardiovascular diseases; HR: Hazard ratio; PY: Person-year.

Only autoimmune diseases with ≥ 20 cardiovascular disease events occurring in the exposed group were included in our analyses.

Models 1 were adjusted for age at follow-up; Models 2 were additionally adjusted for offspring’s calendar year of birth, maternal country of birth, parity, education, income, and marital status in the year of childbirth; Models 3 were adjusted for factors in model 2 and for maternal cardiovascular and psychiatric diseases before childbirth, maternal pre-gestational hypertension, and pre-gestational diabetes.

Table S13. Hazard ratios and 95% confidence intervals for cardiovascular diseases in offspring exposed to maternal autoimmune diseases according to the timing of diagnosis relative to childbirth

| Outcome | Exposure | Non-exposed | Maternal AID ≤ 2 years before birth | | Maternal AID >2 years before birth | |
| --- | --- | --- | --- | --- | --- | --- |
|  |  | Events (Rate)* | Events (Rate)* | HR (95% CI) | Events (Rate)* | HR (95% CI) |
| **Any CVD** | **Any AID** | 37,952 (18.06) | 645 (21.31) | 1.20 (1.17-1.24) | 1663 (20.95) | 1.15 (1.05-1.26) |
|  | **Endocrine autoimmune diseases** | 39,269 (18.12) | 369 (22.27) | 1.21 (1.16-1.26) | 622 (22.78) | 1.22 (1.08-1.39) |
|  | Type 1 Diabetes | 39,702 (18.14) | 108 (24.73) | 1.19 (1.05-1.35) | 247 (25.56) | 1.36 (1.09-1.70) |
|  | Autoimmune thyroiditis | 37,279 (17.95) | 268 (21.82) | 1.20 (1.15-1.25) | 419 (22.04) | 1.20 (1.05-1.39) |
|  | **Inflammatory arthritis** | 40,037 (18.19) | 47 (21.38) | 1.20 (1.11-1.31) | 176 (20.24) | 1.10 (0.83-1.46) |
|  | Rheumatoid arthritis | 39,751 (18.16) | 29 (23.18) | 1.17 (1.05-1.30) | 128 (21.55) | 1.06 (0.74-1.53) |
|  | **Connective tissue autoimmune diseases** | 40,140 (18.19) | 31 (30.33) | 1.39 (1.24-1.56) | 89 (26.19) | 1.61 (1.07-2.42) |
|  | **Skin autoimmune diseases** | 40,007 (18.21) | 73 (19.36) | 1.16 (1.08-1.23) | 180 (16.89) | 1.01 (0.81-1.27) |
|  | Psoriasis vulgaris | 39,166 (18.14) | 61 (18.45) | 1.16 (1.08-1.24) | 156 (16.97) | 0.97 (0.75-1.24) |
|  | **Gastrointestinal autoimmune diseases** | 39,706 (18.18) | 102 (21.88) | 1.13 (1.05-1.21) | 452 (19.91) | 1.07 (0.88-1.30) |
|  | Crohn disease | 39,906 (18.19) | 27 (21.93) | 1.06 (0.93-1.22) | 132 (19.44) | 0.95 (0.67-1.34) |
|  | Ulcerative colitis | 39,773 (18.19) | 53 (22.49) | 1.04 (0.93-1.16) | 163 (19.47) | 1.19 (0.92-1.54) |
|  | Celiac disease | 39,813 (18.17) | 24 (20.07) | 1.24 (1.09-1.41) | 202 (20.63) | 1.14 (0.82-1.59) |
| **Vascular diseases** | **Any AID** | 4872 (2.29) | 97 (3.16) | 1.11 (1.02-1.20) | 205 (2.55) | 1.12 (0.86-1.45) |
|  | **Endocrine autoimmune diseases** | 5027 (2.29) | 63 (3.75) | 1.17 (1.04-1.31) | 84 (3.03) | 1.14 (0.81-1.62) |
|  | Type 1 Diabetes | 5072 (2.29) | 25 (5.63) | 1.38 (1.02-1.86) | 47 (4.77) | 1.78 (1.10-2.88) |
|  | Autoimmune thyroiditis | 4814 (2.29) | 38 (3.05) | 1.11 (0.99-1.25) | 45 (2.33) | 0.94 (0.60-1.48) |
| Arrhythmia | **Any AID** | 8238 (3.88) | 140 (4.57) | 1.19 (1.12-1.27) | 362 (4.50) | 1.21 (1.00-1.47) |
|  | **Endocrine autoimmune diseases** | 8525 (3.89) | 76 (4.53) | 1.16 (1.06-1.26) | 139 (5.02) | 1.35 (1.05-1.75) |
|  | Type 1 Diabetes | 8621 (3.90) | 24 (5.40) | 1.20 (0.92-1.58) | 53 (5.39) | 1.34 (0.83-2.17) |
|  | Autoimmune thyroiditis | 8122 (3.87) | 52 (4.18) | 1.13 (1.03-1.24) | 95 (4.94) | 1.41 (1.06-1.87) |
|  | **Gastrointestinal autoimmune diseases** | 8618 (3.90) | 26 (5.51) | 1.14 (0.98-1.33) | 96 (4.18) | 0.99 (0.65-1.53) |
| **Structural heart diseases** | **Any AID** | 3548 (1.67) | 60 (1.96) | 1.16 (1.05-1.28) | 207 (2.57) | 1.06 (0.79-1.42) |
|  | **Endocrine autoimmune diseases** | 3661 (1.67) | 41 (2.44) | 1.26 (1.11-1.43) | 113 (4.09) | 1.23 (0.85-1.77) |
|  | Type 1 Diabetes | 3697 (1.67) | 21 (4.74) | 1.40 (0.99-1.97) | 72 (7.35) | 2.04 (1.23-3.39) |
|  | Autoimmune thyroiditis | 3499 (1.66) | 23 (1.85) | 1.25 (1.09-1.42) | 55 (2.86) | 1.13 (0.73-1.75) |

AID: Autoimmune disease; CI: Confidence interval; CVD: cardiovascular diseases; HR: Hazard ratio; PY: Person-year.

* Per 10,000 person-years.

Only autoimmune diseases with ≥ 20 cardiovascular disease events occurring in the exposed group were included in our analyses.

Analyses were adjusted for offspring’s calendar year of birth, maternal country of birth, parity, age, education, income, and marital status in the year of childbirth, maternal cardiovascular and psychiatric diseases before childbirth, maternal pre-gestational hypertension, and pre-gestational diabetes.

Table S14. Hazard ratios and 95% confidence intervals for cardiovascular diseases in offspring according to maternal autoimmune diseases diagnosed within three years postpartum

| Outcome | Exposure | Events (per 10,000 PYs) | | Adjusted  HR (95%CI) |
| --- | --- | --- | --- | --- |
|  |  | Exposed | Non-exposed |  |
| **Any CVD** | **Any AID** | 3510 (21.44) | 36,750 (17.95) | 1.20 (1.15-1.24) |
|  | **Endocrine autoimmune diseases** | 1720 (22.50) | 38,540 (18.05) | 1.26 (1.19-1.33) |
|  | Type 1 Diabetes | 418 (25.06) | 39,842 (18.15) | 1.31 (1.15-1.49) |
|  | Autoimmune thyroiditis | 1377 (22.09) | 38,883 (18.09) | 1.25 (1.18-1.33) |
|  | **Inflammatory arthritis** | 333 (21.73) | 39,927 (18.18) | 1.21 (1.09-1.35) |
|  | Reactive arthritis | 74 (19.38) | 40,186 (18.20) | 1.12 (0.89-1.41) |
|  | Rheumatoid arthritis | 222 (22.32) | 40,038 (18.19) | 1.21 (1.05-1.39) |
|  | Ankylosing spondylitis | 55 (24.20) | 40,205 (18.20) | 1.39 (1.07-1.82) |
|  | **Vasculitis** | 107 (25.90) | 40,153 (18.19) | 1.30 (1.07-1.59) |
|  | Henoch-Schonlein purpura | 69 (23.16) | 40,191 (18.20) | 1.14 (0.88-1.47) |
|  | Polymyalgia rheumatica | 21 (37.49) | 40,239 (18.20) | 2.03 (1.31-3.14) |
|  | **Connective tissue autoimmune diseases** | 154 (26.95) | 40,106 (18.18) | 1.40 (1.18-1.65) |
|  | Systemic lupus erythematosus | 80 (30.89) | 40,180 (18.19) | 1.54 (1.22-1.94) |
|  | Sjögren’s disease | 51 (36.14) | 40,209 (18.19) | 1.85 (1.38-2.48) |
|  | **Skin autoimmune diseases** | 460 (19.44) | 39,800 (18.19) | 1.07 (0.97-1.18) |
|  | Psoriasis vulgaris | 396 (19.07) | 39,864 (18.20) | 1.06 (0.95-1.17) |
|  | Vitiligo | 54 (22.36) | 40,206 (18.20) | 1.21 (0.91-1.60) |
|  | **Blood autoimmune diseases** | 51 (18.90) | 40,209 (18.20) | 1.00 (0.75-1.34) |
|  | Idiopathic thrombocytopenic purpura | 45 (21.15) | 40,215 (18.20) | 1.11 (0.81-1.51) |
|  | **Nervous system autoimmune diseases** | 139 (20.65) | 40,121 (18.20) | 1.17 (0.98-1.38) |
|  | Acute disseminated encephalomyelitis | 25 (27.69) | 40,235 (18.20) | 1.61 (1.08-2.41) |
|  | Multiple sclerosis | 95 (19.59) | 40,165 (18.20) | 1.10 (0.89-1.35) |
|  | **Gastrointestinal autoimmune diseases** | 674 (20.03) | 39,586 (18.18) | 1.11 (1.02-1.20) |
|  | Crohn disease | 195 (19.34) | 40,065 (18.20) | 1.05 (0.91-1.21) |
|  | Ulcerative colitis | 271 (19.23) | 39,989 (18.20) | 1.05 (0.93-1.19) |
|  | Celiac disease | 272 (21.15) | 39,988 (18.19) | 1.19 (1.05-1.34) |
|  | **Other autoimmune diseases** | 149 (20.86) | 40,111 (18.20) | 1.11 (0.94-1.31) |
|  | Sarcoidosis | 47 (18.87) | 40,213 (18.20) | 1.03 (0.77-1.38) |
|  | IgA nephropathy | 96 (22.54) | 40,164 (18.20) | 1.19 (0.97-1.46) |
| **Vascular diseases** | **Any AID** | 453 (2.73) | 4721 (2.28) | 1.17 (1.05-1.30) |
|  | **Endocrine autoimmune diseases** | 227 (2.93) | 4947 (2.29) | 1.24 (1.07-1.44) |
|  | Type 1 Diabetes | 82 (4.82) | 5092 (2.29) | 1.59 (1.16-2.17) |
|  | Autoimmune thyroiditis | 155 (2.45) | 5019 (2.31) | 1.15 (0.97-1.35) |
|  | **Inflammatory arthritis** | 44 (2.83) | 5130 (2.31) | 1.24 (0.91-1.68) |
|  | Rheumatoid arthritis | 35 (3.47) | 5139 (2.31) | 1.48 (1.04-2.09) |
|  | **Skin autoimmune diseases** | 63 (2.63) | 5111 (2.31) | 1.12 (0.86-1.46) |
|  | Psoriasis vulgaris | 52 (2.47) | 5122 (2.31) | 1.06 (0.80-1.42) |
|  | **Gastrointestinal autoimmune diseases** | 87 (2.55) | 5087 (2.31) | 1.07 (0.85-1.34) |
|  | Crohn disease | 27 (2.64) | 5147 (2.31) | 1.13 (0.77-1.68) |
|  | Ulcerative colitis | 40 (2.80) | 5134 (2.31) | 1.17 (0.84-1.63) |
|  | Celiac disease | 26 (1.99) | 5148 (2.31) | 0.81 (0.53-1.23) |
| **Arrhythmias** | **Any AID** | 756 (4.56) | 7984 (3.86) | 1.20 (1.11-1.30) |
|  | **Endocrine autoimmune diseases** | 360 (4.65) | 8380 (3.88) | 1.27 (1.13-1.42) |
|  | Type 1 Diabetes | 91 (5.36) | 8649 (3.90) | 1.35 (1.01-1.80) |
|  | Autoimmune thyroiditis | 283 (4.48) | 8457 (3.89) | 1.25 (1.11-1.42) |
|  | **Inflammatory arthritis** | 76 (4.89) | 8664 (3.90) | 1.28 (1.02-1.61) |
|  | Rheumatoid arthritis | 49 (4.86) | 8691 (3.90) | 1.23 (0.92-1.65) |
|  | **Vasculitis** | 28 (6.68) | 8712 (3.90) | 1.55 (1.04-2.29) |
|  | **Connective tissue autoimmune diseases** | 53 (9.13) | 8687 (3.90) | 2.03 (1.52-2.72) |
|  | Systemic lupus erythematosus | 25 (9.48) | 8715 (3.90) | 1.91 (1.23-2.96) |
|  | Sjögren’s disease | 28 (19.51) | 8712 (3.90) | 4.50 (3.01-6.72) |
|  | **Skin autoimmune diseases** | 92 (3.84) | 8648 (3.91) | 0.99 (0.80-1.23) |
|  | Psoriasis vulgaris | 78 (3.71) | 8662 (3.91) | 0.97 (0.77-1.23) |
|  | **Nervous system autoimmune diseases** | 28 (4.11) | 8712 (3.91) | 1.10 (0.75-1.60) |
|  | **Gastrointestinal autoimmune diseases** | 151 (4.43) | 8589 (3.90) | 1.10 (0.93-1.30) |
|  | Crohn disease | 38 (3.73) | 8702 (3.91) | 0.83 (0.59-1.18) |
|  | Ulcerative colitis | 56 (3.93) | 8684 (3.91) | 0.94 (0.71-1.24) |
|  | Celiac disease | 68 (5.22) | 8672 (3.90) | 1.39 (1.09-1.77) |
|  | **Other autoimmune diseases** | 28 (3.87) | 8712 (3.91) | 0.90 (0.62-1.33) |
| **Structural heart diseases** | **Any AID** | 379 (2.28) | 3436 (1.66) | 1.20 (1.07-1.35) |
|  | **Endocrine autoimmune diseases** | 221 (2.85) | 3594 (1.66) | 1.34 (1.14-1.56) |
|  | Type 1 Diabetes | 106 (6.26) | 3709 (1.67) | 2.39 (1.77-3.24) |
|  | Autoimmune thyroiditis | 138 (2.18) | 3677 (1.69) | 1.17 (0.98-1.40) |
|  | **Inflammatory arthritis** | 28 (1.80) | 3787 (1.70) | 1.07 (0.73-1.56) |
|  | **Skin autoimmune diseases** | 48 (2.00) | 3767 (1.70) | 1.15 (0.86-1.55) |
|  | Psoriasis vulgaris | 44 (2.09) | 3771 (1.70) | 1.20 (0.88-1.63) |
|  | **Gastrointestinal autoimmune diseases** | 55 (1.61) | 3760 (1.71) | 0.98 (0.75-1.28) |
|  | Ulcerative colitis | 20 (1.40) | 3795 (1.71) | 0.89 (0.57-1.38) |
|  | Celiac disease | 27 (2.07) | 3788 (1.70) | 1.26 (0.86-1.83) |

AID: Autoimmune disease; CI: Confidence interval; CVD: cardiovascular diseases; HR: Hazard ratio; PY: Person-year.

Only autoimmune diseases with ≥ 20 cardiovascular disease events occurring in the exposed group were included in our analyses.

Analyses were adjusted for offspring’s calendar year of birth, maternal country of birth, parity, age, education, income, and marital status in the year of childbirth, maternal cardiovascular and psychiatric diseases before childbirth, maternal pre-gestational hypertension, and pre-gestational diabetes.

Table S15. Hazard ratios and 95% confidence intervals for cardiovascular diseases in offspring according to maternal autoimmune diseases defined as having at least two diagnosis records for each specific autoimmune disease

| Outcome | Exposure | Events (per 10,000 PYs) | | Adjusted  HR (95% CI) |
| --- | --- | --- | --- | --- |
|  |  | Exposed | Non-exposed |  |
| **Any CVD** | **Any AID** | 1899 (21.91) | 38,361 (18.05) | 1.21 (1.16-1.28) |
|  | **Endocrine autoimmune diseases** | 1034 (23.38) | 39,226 (18.10) | 1.30 (1.21-1.39) |
|  | Type 1 Diabetes | 317 (27.00) | 39,943 (18.16) | 1.37 (1.19-1.59) |
|  | Autoimmune thyroiditis | 760 (22.49) | 39,500 (18.14) | 1.29 (1.20-1.39) |
|  | **Inflammatory arthritis** | 196 (21.68) | 40,064 (18.19) | 1.20 (1.03-1.38) |
|  | Rheumatoid arthritis | 162 (22.94) | 40,098 (18.19) | 1.25 (1.06-1.46) |
|  | Ankylosing spondylitis | 32 (22.09) | 40,228 (18.20) | 1.27 (0.89-1.81) |
|  | **Vasculitis** | 22 (31.10) | 40,238 (18.20) | 1.61 (1.06-2.45) |
|  | **Connective tissue autoimmune diseases** | 103 (28.24) | 40,157 (18.19) | 1.45 (1.19-1.78) |
|  | Systemic lupus erythematosus | 59 (31.91) | 40,201 (18.19) | 1.62 (1.24-2.11) |
|  | Sjögren’s disease | 25 (51.54) | 40,235 (18.20) | 2.42 (1.58-3.71) |
|  | **Skin autoimmune diseases** | 208 (17.27) | 40,052 (18.21) | 0.95 (0.83-1.10) |
|  | Psoriasis vulgaris | 205 (17.21) | 40,055 (18.21) | 0.95 (0.83-1.10) |
|  | **Blood autoimmune diseases** | 23 (20.25) | 40,237 (18.20) | 1.03 (0.67-1.60) |
|  | Idiopathic thrombocytopenic purpura | 23 (22.51) | 40,237 (18.20) | 1.15 (0.74-1.78) |
|  | **Nervous system autoimmune diseases** | 65 (21.15) | 40,195 (18.20) | 1.19 (0.93-1.53) |
|  | Multiple sclerosis | 45 (17.97) | 40,215 (18.21) | 0.99 (0.73-1.35) |
|  | **Gastrointestinal autoimmune diseases** | 307 (21.31) | 39,953 (18.19) | 1.18 (1.05-1.32) |
|  | Crohn disease | 112 (21.22) | 40,148 (18.20) | 1.14 (0.94-1.38) |
|  | Ulcerative colitis | 134 (19.76) | 40,126 (18.20) | 1.07 (0.90-1.28) |
|  | Celiac disease | 75 (23.79) | 40,185 (18.20) | 1.36 (1.08-1.71) |
|  | **Other autoimmune diseases** | 50 (19.59) | 40,210 (18.20) | 1.02 (0.77-1.35) |
|  | IgA nephropathy | 34 (19.89) | 40,226 (18.20) | 1.04 (0.74-1.45) |
| **Vascular diseases** | **Any AID** | 250 (2.84) | 4924 (2.29) | 1.19 (1.03-1.36) |
|  | **Endocrine autoimmune diseases** | 139 (3.10) | 5035 (2.30) | 1.25 (1.03-1.52) |
|  | Type 1 Diabetes | 66 (5.51) | 5108 (2.29) | 1.76 (1.26-2.45) |
|  | Autoimmune thyroiditis | 81 (2.36) | 5093 (2.31) | 1.10 (0.87-1.39) |
|  | **Inflammatory arthritis** | 27 (2.95) | 5147 (2.31) | 1.30 (0.88-1.93) |
|  | Rheumatoid arthritis | 22 (3.07) | 5152 (2.31) | 1.33 (0.86-2.06) |
|  | **Skin autoimmune diseases** | 27 (2.22) | 5147 (2.31) | 0.98 (0.66-1.47) |
|  | Psoriasis vulgaris | 25 (2.08) | 5149 (2.31) | 0.91 (0.60-1.38) |
|  | **Gastrointestinal autoimmune diseases** | 46 (3.15) | 5128 (2.31) | 1.29 (0.94-1.76) |
|  | Ulcerative colitis | 25 (3.64) | 5149 (2.31) | 1.41 (0.91-2.19) |
| **Arrhythmias** | **Any AID** | 401 (4.57) | 8339 (3.88) | 1.20 (1.08-1.34) |
|  | **Endocrine autoimmune diseases** | 224 (5.00) | 8516 (3.89) | 1.39 (1.20-1.61) |
|  | Type 1 Diabetes | 64 (5.35) | 8676 (3.90) | 1.28 (0.93-1.76) |
|  | Autoimmune thyroiditis | 169 (4.94) | 8571 (3.89) | 1.43 (1.22-1.67) |
|  | **Inflammatory arthritis** | 42 (4.59) | 8698 (3.91) | 1.17 (0.86-1.61) |
|  | Rheumatoid arthritis | 37 (5.17) | 8703 (3.90) | 1.31 (0.93-1.83) |
|  | **Connective tissue autoimmune diseases** | 33 (8.91) | 8707 (3.90) | 1.89 (1.31-2.74) |
|  | **Skin autoimmune diseases** | 40 (3.29) | 8700 (3.91) | 0.84 (0.60-1.17) |
|  | Psoriasis vulgaris | 39 (3.24) | 8701 (3.91) | 0.85 (0.61-1.19) |
|  | **Gastrointestinal autoimmune diseases** | 55 (3.77) | 8685 (3.91) | 0.94 (0.71-1.24) |
|  | Crohn disease | 20 (3.74) | 8720 (3.91) | 0.84 (0.52-1.35) |
|  | Ulcerative colitis | 26 (3.79) | 8714 (3.91) | 0.98 (0.66-1.46) |
| **Structural heart diseases** | **Any AID** | 221 (2.52) | 3594 (1.67) | 1.25 (1.08-1.46) |
|  | **Endocrine autoimmune diseases** | 149 (3.32) | 3666 (1.67) | 1.49 (1.23-1.79) |
|  | Type 1 Diabetes | 84 (7.05) | 3731 (1.68) | 2.61 (1.90-3.58) |
|  | Autoimmune thyroiditis | 79 (2.31) | 3736 (1.69) | 1.20 (0.95-1.52) |
|  | **Skin autoimmune diseases** | 25 (2.05) | 3790 (1.70) | 1.21 (0.81-1.81) |
|  | Psoriasis vulgaris | 25 (2.08) | 3790 (1.70) | 1.23 (0.82-1.83) |
|  | **Gastrointestinal autoimmune diseases** | 24 (1.64) | 3791 (1.70) | 1.00 (0.67-1.50) |

AID: Autoimmune disease; CI: Confidence interval; CVD: cardiovascular diseases; HR: Hazard ratio; PY: Person-year.

Only autoimmune diseases with ≥ 20 cardiovascular disease events occurring in the exposed group were included in our analyses.

Analyses were adjusted for offspring’s calendar year of birth, maternal country of birth, parity, age, education, income, and marital status in the year of childbirth, maternal cardiovascular and psychiatric diseases before childbirth, maternal pre-gestational hypertension, and pre-gestational diabetes.

Table S16. Hazard ratios and 95% confidence intervals for cardiovascular diseases in offspring according to maternal exposure to only one autoimmune disease

| Outcome | Exposure | Events (per 10,000 PYs) | | Adjusted  HR (95% CI) |
| --- | --- | --- | --- | --- |
|  |  | Exposed | Non-exposed |  |
| **Any CVD** | **Any AID** | 2374 (21.00) | 37,593 (18.02) | 1.18 (1.13-1.23) |
|  | **Endocrine autoimmune diseases** | 1167 (22.04) | 38,800 (18.08) | 1.24 (1.16-1.32) |
|  | Type 1 Diabetes | 300 (25.17) | 39,667 (18.13) | 1.29 (1.11-1.49) |
|  | Autoimmune thyroiditis | 866 (21.19) | 39,101 (18.12) | 1.23 (1.14-1.32) |
|  | **Inflammatory arthritis** | 167 (19.54) | 39,800 (18.17) | 1.12 (0.96-1.30) |
|  | Reactive arthritis | 32 (15.29) | 39,935 (18.18) | 0.92 (0.65-1.30) |
|  | Rheumatoid arthritis | 113 (21.21) | 39,854 (18.17) | 1.18 (0.97-1.43) |
|  | Ankylosing spondylitis | 22 (19.58) | 39,945 (18.17) | 1.19 (0.78-1.80) |
|  | **Vasculitis** | 87 (27.33) | 39,880 (18.16) | 1.37 (1.09-1.71) |
|  | Henoch-Schonlein purpura | 63 (25.17) | 39,904 (18.16) | 1.25 (0.96-1.63) |
|  | **Connective tissue autoimmune diseases** | 75 (25.33) | 39,892 (18.16) | 1.36 (1.08-1.72) |
|  | Systemic lupus erythematosus | 28 (25.99) | 39,939 (18.17) | 1.38 (0.94-2.03) |
|  | **Skin autoimmune diseases** | 223 (17.67) | 39,744 (18.18) | 1.00 (0.87-1.14) |
|  | Psoriasis vulgaris | 192 (17.39) | 39,775 (18.18) | 0.98 (0.85-1.14) |
|  | Vitiligo | 27 (20.77) | 39,940 (18.17) | 1.19 (0.80-1.76) |
|  | **Blood autoimmune diseases** | 34 (20.02) | 39,933 (18.17) | 1.02 (0.71-1.47) |
|  | Idiopathic thrombocytopenic purpura | 30 (21.01) | 39,937 (18.17) | 1.04 (0.70-1.54) |
|  | **Nervous system autoimmune diseases** | 79 (19.71) | 39,888 (18.17) | 1.13 (0.90-1.41) |
|  | Multiple sclerosis | 48 (17.10) | 39,919 (18.17) | 0.96 (0.71-1.28) |
|  | **Gastrointestinal autoimmune diseases** | 447 (19.69) | 39,520 (18.16) | 1.10 (1.00-1.21) |
|  | Crohn disease | 107 (20.05) | 39,860 (18.17) | 1.12 (0.93-1.36) |
|  | Ulcerative colitis | 159 (19.95) | 39,808 (18.17) | 1.10 (0.93-1.29) |
|  | Celiac disease | 181 (19.33) | 39,786 (18.17) | 1.10 (0.94-1.28) |
|  | **Other autoimmune diseases** | 95 (21.50) | 39,872 (18.17) | 1.19 (0.97-1.45) |
|  | Sarcoidosis | 27 (21.82) | 39,940 (18.17) | 1.28 (0.88-1.87) |
|  | IgA nephropathy | 63 (21.52) | 39,904 (18.17) | 1.17 (0.91-1.50) |
| **Vascular diseases** | **Any AID** | 311 (2.71) | 4827 (2.29) | 1.18 (1.04-1.33) |
|  | **Endocrine autoimmune diseases** | 161 (3.00) | 4977 (2.29) | 1.29 (1.08-1.53) |
|  | Type 1 Diabetes | 65 (5.35) | 5073 (2.29) | 1.77 (1.25-2.48) |
|  | Autoimmune thyroiditis | 96 (2.32) | 5042 (2.31) | 1.15 (0.93-1.42) |
|  | **Inflammatory arthritis** | 20 (2.31) | 5118 (2.31) | 1.06 (0.68-1.66) |
|  | **Skin autoimmune diseases** | 28 (2.20) | 5110 (2.31) | 0.95 (0.64-1.42) |
|  | Psoriasis vulgaris | 24 (2.15) | 5114 (2.31) | 0.90 (0.58-1.40) |
|  | **Gastrointestinal autoimmune diseases** | 63 (2.74) | 5075 (2.30) | 1.20 (0.93-1.56) |
|  | Crohn disease | 20 (3.70) | 5118 (2.30) | 1.62 (1.04-2.55) |
|  | Ulcerative colitis | 26 (3.22) | 5112 (2.30) | 1.32 (0.87-2.01) |
| **Arrhythmias** | **Any AID** | 505 (4.41) | 8167 (3.87) | 1.17 (1.06-1.29) |
|  | **Endocrine autoimmune diseases** | 245 (4.57) | 8427 (3.88) | 1.26 (1.10-1.45) |
|  | Type 1 Diabetes | 70 (5.77) | 8602 (3.89) | 1.41 (1.03-1.94) |
|  | Autoimmune thyroiditis | 175 (4.23) | 8497 (3.89) | 1.23 (1.05-1.44) |
|  | **Inflammatory arthritis** | 36 (4.16) | 8636 (3.90) | 1.13 (0.81-1.57) |
|  | Rheumatoid arthritis | 27 (5.01) | 8645 (3.90) | 1.33 (0.90-1.95) |
|  | **Vasculitis** | 23 (7.11) | 8649 (3.90) | 1.62 (1.04-2.51) |
|  | **Connective tissue autoimmune diseases** | 20 (6.64) | 8652 (3.90) | 1.55 (0.98-2.46) |
|  | **Skin autoimmune diseases** | 42 (3.30) | 8630 (3.90) | 0.87 (0.64-1.20) |
|  | Psoriasis vulgaris | 36 (3.23) | 8636 (3.90) | 0.89 (0.64-1.25) |
|  | **Gastrointestinal autoimmune diseases** | 95 (4.13) | 8577 (3.90) | 1.05 (0.85-1.30) |
|  | Ulcerative colitis | 34 (4.21) | 8638 (3.90) | 1.07 (0.76-1.52) |
|  | Celiac disease | 42 (4.44) | 8630 (3.90) | 1.17 (0.85-1.60) |
|  | **Other autoimmune diseases** | 20 (4.47) | 8652 (3.90) | 1.14 (0.73-1.77) |
| **Structural heart diseases** | **Any AID** | 268 (2.34) | 3513 (1.66) | 1.25 (1.09-1.43) |
|  | **Endocrine autoimmune diseases** | 155 (2.89) | 3626 (1.67) | 1.37 (1.14-1.65) |
|  | Type 1 Diabetes | 71 (5.87) | 3710 (1.68) | 2.13 (1.52-2.99) |
|  | Autoimmune thyroiditis | 83 (2.01) | 3698 (1.69) | 1.12 (0.89-1.41) |
|  | **Skin autoimmune diseases** | 27 (2.12) | 3754 (1.70) | 1.23 (0.83-1.83) |
|  | Psoriasis vulgaris | 26 (2.33) | 3755 (1.70) | 1.36 (0.91-2.03) |
|  | **Gastrointestinal autoimmune diseases** | 36 (1.56) | 3745 (1.70) | 1.00 (0.72-1.40) |

AID: Autoimmune disease; CI: Confidence interval; CVD: cardiovascular diseases; HR: Hazard ratio; PY: Person-year.

Only autoimmune diseases with ≥ 20 cardiovascular disease events occurring in the exposed group were included in our analyses.

Analyses were adjusted for offspring’s calendar year of birth, maternal country of birth, parity, age, education, income, and marital status in the year of childbirth, maternal cardiovascular and psychiatric diseases before childbirth, maternal pre-gestational hypertension, and pre-gestational diabetes.

Table S17. Hazard ratios and 95% confidence intervals for cardiovascular diseases in offspring according to maternal autoimmune diseases defined as having at least two diagnosis records for each specific cardiovascular disease

| Outcome | Exposure | Events (per 10,000 PYs) | | Adjusted  HR (95% CI) |
| --- | --- | --- | --- | --- |
|  |  | Exposed | Non-exposed |  |
| **Any CVD** | **Any AID** | 1205 (9.55) | 17,198 (8.19) | 1.19 (1.11-1.26) |
|  | **Endocrine autoimmune diseases** | 607 (10.24) | 17,796 (8.21) | 1.26 (1.15-1.38) |
|  | Type 1 Diabetes | 182 (12.25) | 18,221 (8.24) | 1.26 (1.03-1.53) |
|  | Autoimmune thyroiditis | 457 (9.89) | 17,946 (8.23) | 1.29 (1.17-1.42) |
|  | **Inflammatory arthritis** | 99 (8.78) | 18,304 (8.26) | 1.09 (0.89-1.34) |
|  | Rheumatoid arthritis | 74 (9.99) | 18,329 (8.26) | 1.21 (0.95-1.53) |
|  | **Vasculitis** | 45 (11.73) | 18,358 (8.26) | 1.31 (0.96-1.78) |
|  | Henoch-Schonlein purpura | 30 (10.37) | 18,373 (8.26) | 1.20 (0.82-1.75) |
|  | **Connective tissue autoimmune diseases** | 71 (15.45) | 18,332 (8.25) | 1.80 (1.42-2.29) |
|  | Systemic lupus erythematosus | 38 (18.16) | 18,365 (8.26) | 2.06 (1.47-2.86) |
|  | Sjögren’s disease | 24 (26.97) | 18,379 (8.26) | 3.21 (2.11-4.88) |
|  | **Skin autoimmune diseases** | 100 (6.8) | 18,303 (8.28) | 0.88 (0.71-1.07) |
|  | Psoriasis vulgaris | 88 (6.91) | 18,315 (8.27) | 0.88 (0.71-1.10) |
|  | **Nervous system autoimmune diseases** | 39 (8.27) | 18,364 (8.27) | 1.05 (0.76-1.45) |
|  | Multiple sclerosis | 21 (6.33) | 18,382 (8.27) | 0.77 (0.49-1.22) |
|  | **Gastrointestinal autoimmune diseases** | 249 (8.88) | 18,154 (8.26) | 1.11 (0.98-1.26) |
|  | Crohn disease | 71 (8.56) | 18,332 (8.27) | 1.04 (0.82-1.32) |
|  | Ulcerative colitis | 101 (9.09) | 18,302 (8.26) | 1.12 (0.92-1.37) |
|  | Celiac disease | 94 (8.41) | 18,309 (8.27) | 1.09 (0.88-1.34) |
|  | **Other autoimmune diseases** | 48 (8.92) | 18,355 (8.26) | 1.06 (0.79-1.41) |
|  | IgA nephropathy | 31 (8.76) | 18,372 (8.27) | 1.04 (0.73-1.48) |
| **Vascular diseases** | **Any AID** | 219 (1.72) | 3267 (1.55) | 1.10 (0.95-1.28) |
|  | **Endocrine autoimmune diseases** | 113 (1.89) | 3373 (1.55) | 1.24 (1.00-1.53) |
|  | Type 1 Diabetes | 43 (2.87) | 3443 (1.55) | 1.65 (1.08-2.53) |
|  | Autoimmune thyroiditis | 76 (1.63) | 3410 (1.56) | 1.16 (0.91-1.47) |
|  | **Skin autoimmune diseases** | 20 (1.35) | 3466 (1.56) | 0.80 (0.49-1.31) |
|  | **Gastrointestinal autoimmune diseases** | 47 (1.67) | 3439 (1.56) | 1.07 (0.79-1.44) |
|  | Ulcerative colitis | 23 (2.06) | 3463 (1.55) | 1.23 (0.78-1.93) |
| **Arrhythmias** | **Any AID** | 337 (2.66) | 4694 (2.22) | 1.22 (1.08-1.37) |
|  | **Endocrine autoimmune diseases** | 163 (2.73) | 4868 (2.23) | 1.31 (1.10-1.55) |
|  | Type 1 Diabetes | 42 (2.80) | 4989 (2.24) | 1.14 (0.76-1.72) |
|  | Autoimmune thyroiditis | 129 (2.78) | 4902 (2.24) | 1.37 (1.14-1.65) |
|  | **Inflammatory arthritis** | 26 (2.29) | 5005 (2.25) | 1.01 (0.68-1.51) |
|  | Rheumatoid arthritis | 20 (2.68) | 5011 (2.25) | 1.15 (0.72-1.82) |
|  | **Connective tissue autoimmune diseases** | 30 (6.47) | 5001 (2.24) | 2.57 (1.76-3.75) |
|  | **Skin autoimmune diseases** | 22 (1.49) | 5009 (2.25) | 0.72 (0.47-1.10) |
|  | Psoriasis vulgaris | 20 (1.56) | 5011 (2.25) | 0.75 (0.48-1.18) |
|  | **Gastrointestinal autoimmune diseases** | 66 (2.34) | 4965 (2.25) | 1.00 (0.78-1.29) |
|  | Crohn disease | 27 (2.42) | 5004 (2.25) | 1.05 (0.71-1.55) |
|  | Ulcerative colitis | 26 (2.31) | 5005 (2.25) | 1.07 (0.72-1.59) |
| **Structural heart diseases** | **Any AID** | 180 (1.42) | 2326 (1.10) | 1.09 (0.92-1.29) |
|  | **Endocrine autoimmune diseases** | 114 (1.91) | 2392 (1.10) | 1.30 (1.04-1.61) |
|  | Type 1 Diabetes | 62 (4.14) | 2444 (1.10) | 2.53 (1.72-3.74) |
|  | Autoimmune thyroiditis | 64 (1.38) | 2442 (1.11) | 1.08 (0.83-1.40) |
|  | **Gastrointestinal autoimmune diseases** | 31 (1.10) | 2475 (1.12) | 1.05 (0.73-1.49) |

AID: Autoimmune disease; CI: Confidence interval; CVD: cardiovascular diseases; HR: Hazard ratio; PY: Person-year.

Only autoimmune diseases with ≥ 20 cardiovascular disease events occurring in the exposed group were included in our analyses.

Analyses were adjusted for offspring’s calendar year of birth, maternal country of birth, parity, age, education, income, and marital status in the year of childbirth, maternal cardiovascular and psychiatric diseases before childbirth, maternal pre-gestational hypertension, and pre-gestational diabetes.

Table S18. Hazard ratios and 95% confidence intervals for cardiovascular diseases in offspring according to maternal autoimmune diseases in sensitivity analysis using alternative definitions to identify specific cardiovascular diseases

| Outcome | Exposure | Events (per 10,000 PYs) | | Adjusted  HR (95% CI) |
| --- | --- | --- | --- | --- |
|  |  | Exposed | Non exposed |  |
| **Vascular diseases** | **Any AID** | 302 (2.38) | 4190 (1.98) | 1.19 (1.05-1.35) |
|  | **Endocrine autoimmune diseases** | 157 (2.63) | 4335 (1.99) | 1.30 (1.08-1.56) |
|  | Type 1 Diabetes | 70 (4.67) | 4422 (1.99) | 1.89 (1.33-2.67) |
|  | Autoimmune thyroiditis | 94 (2.02) | 4398 (2.01) | 1.13 (0.91-1.40) |
|  | **Inflammatory arthritis** | 26 (2.29) | 4466 (2.00) | 1.18 (0.79-1.77) |
|  | Rheumatoid arthritis | 22 (2.95) | 4470 (2.00) | 1.49 (0.96-2.31) |
|  | **Skin autoimmune diseases** | 30 (2.03) | 4462 (2.01) | 1.04 (0.71-1.53) |
|  | Psoriasis vulgaris | 24 (1.88) | 4468 (2.01) | 0.92 (0.59-1.42) |
|  | **Gastrointestinal autoimmune diseases** | 64 (2.27) | 4428 (2.00) | 1.15 (0.89-1.50) |
|  | Crohn disease | 22 (2.64) | 4470 (2.00) | 1.37 (0.89-2.10) |
|  | Ulcerative colitis | 28 (2.51) | 4464 (2.00) | 1.22 (0.81-1.82) |
| **Arrhythmias** | **Any AID** | 573 (4.52) | 8167 (3.87) | 1.19 (1.08-1.30) |
|  | **Endocrine autoimmune diseases** | 282 (4.73) | 8458 (3.89) | 1.30 (1.14-1.48) |
|  | Type 1 Diabetes | 82 (5.47) | 8658 (3.90) | 1.35 (1.00-1.83) |
|  | Autoimmune thyroiditis | 211 (4.55) | 8529 (3.90) | 1.30 (1.12-1.50) |
|  | **Inflammatory arthritis** | 47 (4.15) | 8693 (3.91) | 1.08 (0.80-1.45) |
|  | Rheumatoid arthritis | 38 (5.10) | 8702 (3.91) | 1.29 (0.93-1.80) |
|  | **Vasculitis** | 24 (6.21) | 8716 (3.91) | 1.40 (0.91-2.16) |
|  | **Connective tissue autoimmune diseases** | 39 (8.43) | 8701 (3.90) | 1.88 (1.34-2.63) |
|  | Systemic lupus erythematosus | 21 (9.94) | 8719 (3.90) | 2.04 (1.27-3.29) |
|  | **Skin autoimmune diseases** | 48 (3.25) | 8692 (3.91) | 0.83 (0.62-1.13) |
|  | Psoriasis vulgaris | 42 (3.29) | 8698 (3.91) | 0.87 (0.63-1.20) |
|  | **Gastrointestinal autoimmune diseases** | 123 (4.37) | 8617 (3.90) | 1.08 (0.89-1.30) |
|  | Crohn disease | 32 (3.84) | 8708 (3.91) | 0.87 (0.59-1.26) |
|  | Ulcerative colitis | 46 (4.12) | 8694 (3.91) | 0.97 (0.71-1.33) |
|  | Celiac disease | 55 (4.90) | 8685 (3.90) | 1.30 (0.99-1.71) |
|  | **Other autoimmune diseases** | 22 (4.07) | 8718 (3.91) | 0.99 (0.64-1.51) |
| **Structural heart diseases** | **Any AID** | 182 (1.43) | 2131 (1.01) | 1.24 (1.05-1.46) |
|  | **Endocrine autoimmune diseases** | 108 (1.81) | 2205 (1.01) | 1.36 (1.09-1.70) |
|  | Type 1 Diabetes | 54 (3.61) | 2259 (1.02) | 1.98 (1.33-2.96) |
|  | Autoimmune thyroiditis | 64 (1.38) | 2249 (1.03) | 1.24 (0.96-1.61) |
|  | **Gastrointestinal autoimmune diseases** | 28 (0.99) | 2285 (1.03) | 1.01 (0.70-1.47) |

AID: Autoimmune disease; CI: Confidence interval; HR: Hazard ratio; PY: Person-year.

Vascular diseases were identified using information on primary diagnoses in the Swedish Patient Register (only inpatient) and information of main cause of death from the Swedish Cause of Death Register.

Arrhythmias were identified using information on both primary and secondary diagnoses in the Swedish Patient Register (both inpatient and outpatient).

Structural heart diseases were identified using information on primary diagnoses in the Swedish Patient Register (both inpatient and outpatient).

Only autoimmune diseases with ≥ 20 cardiovascular disease events occurring in the exposed group were included in our analyses.

Analyses were adjusted for offspring’s calendar year of birth, maternal country of birth, parity, age, education, income, and marital status in the year of childbirth, maternal cardiovascular and psychiatric diseases before childbirth, maternal pre-gestational hypertension, and pre-gestational diabetes.

Table S19. E-values for the associations between maternal autoimmune diseases and cardiovascular diseases in offspring

| Disease | HR | CI lower | CI upper | E-value |  |
| --- | --- | --- | --- | --- | --- |
|  |  |  |  | HR | CI lower |
| Any AID | 1.19 | 1.14 | 1.24 | 1.67 | 1.54 |
| Endocrine autoimmune diseases | 1.25 | 1.18 | 1.33 | 1.81 | 1.64 |
| Inflammatory arthritis | 1.17 | 1.03 | 1.34 | 1.62 | 1.21 |
| Vasculitis | 1.29 | 1.05 | 1.6 | 1.90 | 1.28 |
| Connective tissue autoimmune diseases | 1.45 | 1.21 | 1.74 | 2.26 | 1.71 |
| Skin autoimmune diseases | 0.98 | 0.86 | 1.12 | NA | NA |
| Blood autoimmune diseases | 1.04 | 0.75 | 1.43 | 1.24 | NA |
| Nervous system autoimmune diseases | 1.15 | 0.93 | 1.41 | 1.56 | NA |
| Gastrointestinal autoimmune diseases | 1.12 | 1.03 | 1.22 | 1.49 | 1.21 |
| Other autoimmune diseases | 1.17 | 0.97 | 1.4 | 1.62 | NA |

AID: Autoimmune disease; CI: Confidence interval; HR: Hazard ratio.

The E-values were calculated based on hazard ratio estimates (Model 3) from Figure 2.

Model 3 were adjusted for offspring’s calendar year of birth, maternal country of birth, parity, age, education, income, and marital status in the year of childbirth, maternal cardiovascular and psychiatric diseases before childbirth, maternal pre-gestational hypertension, and pre-gestational diabetes. NA: hazard ratio < 1.

Figure S1. Directed acyclic graph illustrating assumed causal relationships between maternal autoimmune disease and offspring cardiovascular disease.

Pink nodes represent pre-exposure confounders adjusted for in the main models (maternal age, calendar year, country of birth, education, income, parity, marital status). Nodes in Model 3 (maternal CVD and psychiatric disorders before childbirth, and pre-gestational diabetes, pre-gestational hypertension) may lie on the causal pathway and are displayed separately to avoid overadjustment. Blue nodes represent mediators occurring after exposure and used in causal mediation analyses (preterm birth, SGA, LGA, CHD). Gray dashed nodes represent unmeasured shared familial factors.

AID: Autoimmune disease; CHD: congenital heart disease; CVD: Cardiovascular disease; LGA: Large for gestational age; SGA: Small for gestational age.

Figure S2. Log-minus-log survival curves of offspring by maternal autoimmune disorder status.

AID: Autoimmune disease.

Figure S3. Directed acyclic graph illustrating assumptions for the causal mediation analysis. Maternal age, education, income, country of birth, parity, marital status at delivery, calendar year, and psychiatric disorders before childbirth were treated as mediator-outcome confounders and adjusted for in the mediation models. Gray dashed nodes represent unmeasured shared familial factors and potential mediator-outcome confounders (e.g., gestational weight gain, alcohol consumption, medication use during pregnancy) that could bias natural direct and indirect effect estimates if present.

Figure S4. Adjusted hazard ratios and 95% confidence intervals for offspring cardiovascular diseases over time associated with maternal autoimmune diseases

AID: Autoimmune diseases; CI: Confidence interval.

Analyses were adjusted for offspring’s calendar year of birth, maternal country of birth, parity, age, education, income, and marital status in the year of childbirth, maternal cardiovascular and psychiatric diseases before childbirth, maternal pre-gestational hypertension, and pre-gestational diabetes.

Figure S5. Hazard ratios and 95% confidence intervals for cardiovascular diseases in offspring according to any maternal autoimmune disease, stratified by offspring’s sex

CI: Confidence interval; CVD: cardiovascular diseases; PY: Person-year.

Analyses were adjusted for offspring’s calendar year of birth, maternal country of birth, parity, age, education, income, and marital status in the year of childbirth, maternal cardiovascular and psychiatric diseases before childbirth, maternal pre-gestational hypertension, and pre-gestational diabetes.

Figure S6. Hazard ratios and 95% confidence intervals for cardiovascular diseases in offspring according to any maternal autoimmune disease, stratified by offspring’s age

CI: Confidence interval; CVD: cardiovascular diseases; PY: Person-year.

Younger: follow-up years <10; Older: follow-up years ≥10.

Analyses were adjusted for offspring’s calendar year of birth, maternal country of birth, parity, age, education, income, and marital status in the year of childbirth, maternal cardiovascular and psychiatric diseases before childbirth, maternal pre-gestational hypertension, and pre-gestational diabetes.

Figure S7. Hazard ratios and 95% confidence intervals for cardiovascular diseases in offspring according to any maternal autoimmune disease, stratified by income

CI: Confidence interval; CVD: cardiovascular diseases; PY: Person-year.

Analyses were adjusted for offspring’s calendar year of birth, maternal country of birth, parity, age, education, and marital status in the year of childbirth, maternal cardiovascular and psychiatric diseases before childbirth, maternal pre-gestational hypertension, and pre-gestational diabetes.

Figure S8. Hazard ratios and 95% confidence intervals for cardiovascular diseases in offspring according to any maternal autoimmune disease, stratified by maternal cardiovascular diseases

CI: Confidence interval; CVD: cardiovascular diseases; PY: Person-year.

The “No CVD” group includes offspring who were not exposed to maternal cardiovascular diseases before childbirth; the “CVD” group includes offspring exposed to any maternal cardiovascular diseases before childbirth.

Analyses were adjusted for offspring’s calendar year of birth, maternal country of birth, parity, age, education, income, and marital status in the year of childbirth, maternal psychiatric diseases before childbirth, maternal pre-gestational hypertension, and pre-gestational diabetes.

Figure S9. Hazard ratios and 95% confidence intervals for cardiovascular diseases in offspring according to any maternal autoimmune disease, stratified by maternal hypertension

CI: Confidence interval; CVD: cardiovascular diseases; HP: Hypertension; PY: Person-year.

The “No HP” group includes offspring who were not exposed to maternal hypertensive disorders before childbirth; the “HP” group includes offspring exposed to maternal hypertensive disorders before childbirth.

Analyses were adjusted for offspring’s calendar year of birth, maternal country of birth, parity, age, education, income, and marital status in the year of childbirth, maternal cardiovascular and psychiatric diseases before childbirth, and pre-gestational diabetes.

Figure S10. Hazard ratios and 95% confidence intervals for cardiovascular diseases in offspring according to any maternal autoimmune disease, stratified by maternal diabetes

CI: Confidence interval; CVD: cardiovascular diseases; DM: Diabetes mellitus; PY: Person-year.

The “No DM” group includes offspring who were not exposed to maternal diabetes before childbirth; the “DM” group includes offspring exposed to maternal diabetes before childbirth.

Analyses were adjusted for offspring’s calendar year of birth, maternal country of birth, parity, age, education, income, and marital status in the year of childbirth, maternal cardiovascular and psychiatric diseases before childbirth, and maternal pre-gestational hypertension.
